# Feasibility of the Olympic marathon under climatic and socioeconomic change

**DOI:** 10.1038/s41598-022-07934-6

**Published:** 2022-03-07

**Authors:** Takahiro Oyama, Jun’ya Takakura, Minoru Fujii, Kenichi Nakajima, Yasuaki Hijioka

**Affiliations:** 1 Department of Environment Systems, Graduate School of Frontier Sciences, The University of Tokyo, 5-1-5, Kashiwanoha, Kashiwa, 277-8561 Japan; 2 Social Systems Division, National Institute for Environmental Studies, 16-2, Onogawa, Tsukuba, 305-8506 Japan; 3 Material Cycles Division, National Institute for Environmental Studies, 16-2, Onogawa, Tsukuba, 305-8506 Japan; 4 Center for Climate Change Adaptation, National Institute for Environmental Studies, 16-2, Onogawa, Tsukuba, 305-8506 Japan

**Keywords:** Climate-change adaptation, Climate-change impacts, Socioeconomic scenarios, Risk factors

## Abstract

There are concerns about the impact of climate change on Olympic Games, especially endurance events, such as marathons. In recent competitions, many marathon runners dropped out of their races due to extreme heat, and it is expected that more areas will be unable to host the Games due to climate change. Here, we show the feasibility of the Olympic marathon considering the variations in climate factors, socioeconomic conditions, and adaptation measures. The number of current possible host cities will decline by up to 27% worldwide by the late twenty-first century. Dozens of emerging cities, especially in Asia, will not be capable of hosting the marathon under the highest emission scenario. Moving the marathon from August to October and holding the Games in multiple cities in the country are effective measures, and they should be considered if we are to maintain the regional diversity of the Games.

## Introduction

The global land temperature in 2011–2020 increased by 1.59 °C from 1850 to 1900 and is projected to increase throughout the twenty-first century^1^. Heat stress, which is a stress effect on an organism that results from exposure to excessive ambient temperatures^2^, has become apparent in humans and is expected to increase further. During 1991–2018, 37% of heat-related deaths in 732 locations across all continents were attributable to anthropogenic climate change^3^. Population exposure to “deadly heat” (wet bulb temperatures above 32 °C) may increase by a factor of five to ten by 2070–2080 compared with 2020^4^, and the number of excess global temperature-related deaths is projected to be 83 million with the 4.1 °C rise by 2100^5^. While elderly individuals and children are particularly vulnerable to heat stress, individuals competing in outdoor sports may also be at risk^6^. Tennis matches were suspended during the 2014 Australian Open after temperatures exceeded 43°C^7^, and 40 of 68 female marathon runners dropped out at a temperature of 33 °C with 73 percent humidity in the 2019 IAAF World Championships in Doha^8^. High air temperatures and elevated humidity negatively affected the performance of Olympic marathon runners in 1896–2004^9^.

The marathon is one of the most symbolic events of the modern Summer Olympic Games (hereinafter called the Games), which are the most watched and most expensive event on earth^10^; thus, the feasibility of the marathon in the Games has important implications both for the health of athletes and the culture of modern humanity. Smith et al.^11^ revealed that few cities outside of Western Europe will be viable to hold the Olympic marathon by 2085 under the Representative Concentration Pathway (RCP) 8.5 based on the wet bulb globe temperature (WBGT)^12^. DeChano-Cook and Shelley^7^ pointed out that many historical host cities of the summer and winter Games will no longer be suitable because of the increases in temperature and sea level.

The purpose of this study is to explain the feasibility of hosting the Olympic marathon more comprehensively, considering the variations in climate change projections, the hourly climatic characteristics of cities, future socioeconomic conditions, and adaptation measures (AMs) that have not been adequately considered in previous studies. We hypothesize that these climatic, socioeconomic, and adaptation factors all have distinctive impacts on the feasibility of hosting an Olympic marathon, and this study quantifies these impacts. We consider the variation in climate change by projecting the future WBGT in August in the mid- and late twenty-first centuries (2040–2059 and 2080–2099) using seven global climate models (GCMs; namely, GFDL_ESM2M, HadGEM2_ES, IPSL_CM5A_LR, MIROC5, MIROC_ESM, MRI_CGCM3, and NorESM1_M) and four emission scenarios (RCP 2.6, 4.5, 6.0, and 8.5)^13^. Since an increase in the WBGT has a negative impact on the performance of most marathon runners including elite athletes^14,15^, and since the WBGT standards suggested by the International Institute for Running Medicine (IIRM)^16^ and American College of Sports Medicine (ACSM)^17^ are referred to in the management of various marathons, we adopt the WBGT as an indicator of the feasibility of hosting an Olympic marathon. We utilize the seven GCMs included in the S14 retrospective meteorological forcing dataset (S14FD)^18^, which was developed based on the Coupled Model Intercomparison Project Phase 5 (CMIP5)^19^. We reproduce hourly urban climatic characteristics by applying a bias correction using meteorological observation data based on the method of Takakura et al.^20^. We consider future socioeconomic conditions using the parameters of population^21,22^, the gross domestic product (GDP)^23^, and its growth rate in the 2030–2050 period and the 2070–2090 period, both are 10 years before the evaluation periods, based on the five Shared Socioeconomic Pathways (SSPs)^24^. We quantify the effects of AMs that have been adopted or considered in recent Games, such as holding the marathon late at night or early in the morning. The main text presents the results for the late twenty-first century (2080–2099), while the results for the mid-twenty-first century (2040–2059) are presented in the Supplementary Information (SI).

## Results

### Climate change impacts under current socioeconomic conditions

First, to clarify the impact of climate change alone, we evaluated the number of cities that could host the Olympic marathon in August during the late twenty-first century (2080–2099) under the socioeconomic conditions in 2020, changing only the climatic conditions according to the four RCPs. A total of 70 cities in 25 countries were selected based on (1) socioeconomic conditions as of 2010 (an urban population of 2.5 million or more, a national GDP of 300 billion dollars or more (purchasing power parity (PPP), Int’l $ 2005) as of 2010 and a GDP growth rate above 0% as of 2010–2015); (2) an elevation of less than 1,600 m^11^; and (3) the availability of meteorological data for WBGT correction using the method of Takakura et al.^20^. See the Methods section for the rationale behind the selection criteria above.

As evaluation criteria for cities, the WBGT levels were set as follows based on the four alert levels (low, moderate, high, and extreme) in the IIRM Medical Care manual^16^.

WBGT level 1 (good): There is a greater than 90% probability that the WBGT will fall below 18°C for at least three consecutive hours between 7:00 and 21:00 in August. WBGT level 1 corresponds to a low alert level and “good conditions” in the IIRM's manual.

WBGT level 2 (caution): Level 1 does not apply, and there is a greater than 90% probability that the WBGT will fall below 22°C for at least 3 consecutive hours between 7:00 and 21:00 in August. WBGT level 2 corresponds to a moderate alert level and “less than ideal conditions” in the IIRM's manual.

WBGT level 3 (warning): Levels 1 and 2 do not apply, and there is a greater than 90% probability that the WBGT will fall below 28°C for at least 3 consecutive hours between 7:00 and 21:00 in August. WBGT level 3 corresponds to a high alert level and “potentially dangerous conditions” in the IIRM's manual.

WBGT level 4 (cancel): Levels 1, 2, and 3 do not apply. WBGT level 4 corresponds to an extreme alert level and “event cancelled / extreme and dangerous conditions” in the IIRM's manual.

We determine that cities with WBGT levels of 1 to 3 can host the Olympic marathon, while those with a WBGT level of 4 cannot. It has recently been found that the performance of elite marathon runners improves as the dry bulb temperature rises^25^, which is closely related to the WBGT. However, there would have been few cases where the WBGT exceeded 28 °C in the Berlin Marathon races covered in the study even momentarily, based on the dry bulb temperature range in the races. Therefore, the use of WBGT Level 4 (28 °C) as a threshold for the impossibility of holding a marathon is consistent with the results of the aforementioned research. The 90% criterion is set in reference to previous studies on the feasibility of hosting the summer and winter Olympics^11,26,27^, and the duration (3 h) and timing (between 7:00 and 21:00 in August) is set based on the general competition time of the Olympic marathon, where all Olympic marathons since the 1980 Moscow Olympics were held, except for the men's and women's marathons at the 1988 Seoul and 2000 Sydney Olympics, the women's marathon at the 1996 Atlanta Olympics, and the women's marathon at the 2020 Tokyo Olympics, which was moved up by an hour the day before to start at 6:00^28–33^.

**Figure 1:**
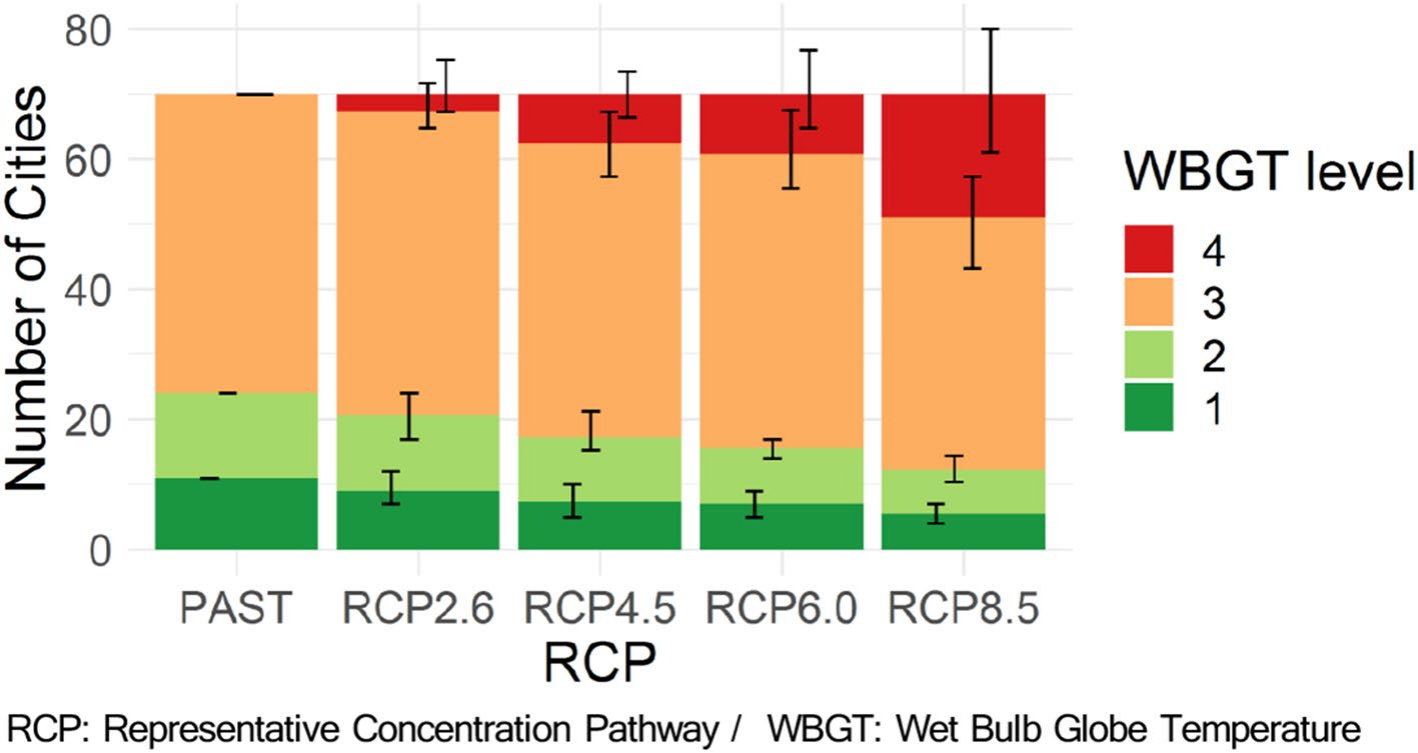
The WBGT levels of 70 cities in August in the late-twenty-first century (2080–2099). The cities are selected under the socioeconomic conditions in 2010. The bars represent the average of the seven GCMs. Error bars indicate the range between the maximum and minimum values of the seven GCMs.

As a result, globally, the number of cities that can host the Olympic marathon (WBGT levels 1–3) significantly decreases as the amount of greenhouse gas emissions increases toward the late twenty-first century (2080–2099) (Fig. 1). Under historical (1994–2013) climate conditions, all cities (70 cities) can host the Olympic marathon, while the number of cities decreases to 67.3 (96%) under RCP2.6 and to 51.0 (73%) under RCP8.5 in 2080–2099. The number of safer cities with WBGT levels 1 to 2 decreased from 24 (34%) under historical climate conditions to 20.7 (30%) under RCP2.6 and to 12.3 (18%) under RCP8.5. The error bars indicate the range between the maximum and minimum values of the seven GCMs, and the ranges are large enough to be considered. Therefore, under the current situation where it is not possible to judge the superiority of a particular GCM, the results based on various GCMs, as shown in this paper, are more reliable. The detailed number of cities, including the upper and lower limits by the seven GCMs, is given in Supplementary Table 1. The results for the mid-twenty-first century (2040–2059) are shown in Supplementary Fig. 1 and Supplementary Table 2.

**Figure 2:**
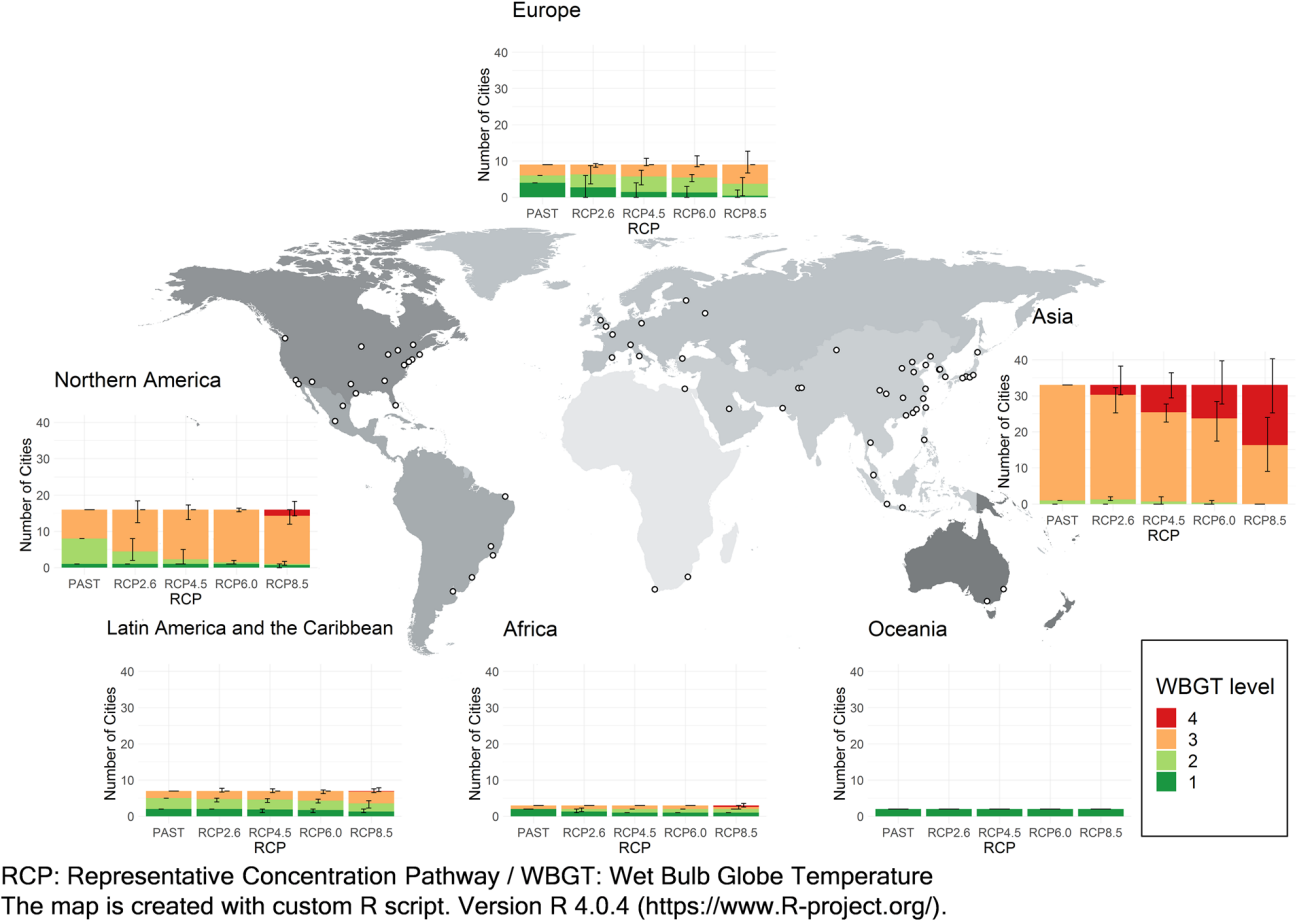
The WBGT levels of 70 cities in August in the late-twenty-first century (2080–2099) by region. The cities are selected under the socioeconomic conditions in 2010. The bars represent the average of the seven GCMs. Error bars indicate the range between the maximum and minimum values of the seven GCMs.

Regionally, there are three patterns in the decrease in the number of cities that can host the Olympic marathon (Fig. 2). The number of cities by country is shown in Supplementary Fig. 3 and the result for the mid-twenty-first century (2040–2059) is shown in Supplementary Fig. 2.

### Pattern 1: Asia

The number of cities that can host the Olympic marathon decreases significantly as the amount of greenhouse gas emissions increases. Among the viable cities, the number of cities with a relatively low heat risk (WBGT levels 1–2) is very small and will decrease further with climate change. This result may be because many cities in Asia are in the mid-latitude zone of the Northern Hemisphere and are affected by the Asian monsoon, resulting in hot and humid summers.

Asia: This region contains the largest number of cities (33) out of the 70 total cities. Under historical (1994–2013) climate conditions, all cities (33 cities) can host the marathon, while the number of cities decreases to 30.3 (92%) under RCP2.6 and to 16.3 (49%) under RCP8.5. The number of cities with a relatively low heat risk (WBGT levels 1–2) decreases from 1 (3%) under historical climate conditions to none under RCP8.5.

### Pattern 2: North America, Latin America and the Caribbean, and Africa

The number of cities that can host the event (WBGT levels 1–3) only slightly decreases even under the highest emission scenario, while the number of cities with a relatively low heat risk (WBGT levels 1–2) will decrease due to climate change. This finding may be because many cities are in the high latitudes of the Northern Hemisphere (North America) or the Southern Hemisphere (Latin America and the Caribbean, and Africa) and are thus less likely to be hot and humid in August than Pattern 1 (Asia).

North America: This region contains the second largest number of cities (16 cities). Under historical (1994–2013) climate conditions, all cities can host the marathon. Under RCP8.5, the number of cities decreases to 15.3 (96%), but under the other scenarios, the number of cities does not change at all or remains mostly unchanged. The number of cities with relatively low heat risk (WBGT levels 1–2) decreases from 8 (50%) under historical climate conditions to 4.4 (28%) under RCP2.6 and to 1.0 (6%) under RCP8.5.

Latin America and the Caribbean: This region contains the third-smallest number of cities (7 cities). Under historical (1994–2013) climate conditions, all cities can host the marathon. The number of cities does not change at all or only slightly decreases under the four RCP scenarios. The number of cities with a relatively low heat risk (WBGT level 1–2) decreased from 5 (71%) under historical climate conditions to 3.6 (51%) under RCP8.5.

Africa: The region contains the second-smallest number of 3 target cities. Under historical (1994–2013) climate conditions, all cities can host the Olympic marathon. The number of cities decreases to 2.6 (87%) under RCP8.5, but under the other scenarios, the number does not change at all or remains mostly unchanged. The number of cities with a relatively low heat risk (WBGT levels 1–2) does not decrease even under RCP8.5, however, the number of cities with WBGT level 1 decreases from 2 (66%) to 1 (33%) under RCP8.5.

### Pattern 3: Europe and Oceania

The number of cities that can host the Olympic marathon does not decrease even under the highest emission scenario. There were originally many cities with a relatively low heat risk (WBGT levels 1–2), which will be the case under future climate conditions. This finding may be because many cities are in the high latitudes of the Northern Hemisphere (Europe) or in the Southern Hemisphere (Oceania), where the WBGT is unlikely to be high in August.

Europe: This region contains the third largest number of target cities, 9. Under historical (1994–2013) climate conditions, all cities can host the Olympic marathon, and under all RCP scenarios, the number does not change at all. The number of cities with a relatively low heat risk (WBGT levels 1–2) decreases from 6 (67%) under historical climate conditions to 3.7 (41%) under RCP8.5.

Oceania: This region contains the smallest number of target cities, 2. Even under the highest emission scenario, the number of cities that can host the Olympics does not decrease, and all four cities are determined to be safe (WBGT level 1) under all RCP scenarios.

### Climate change impacts under future socioeconomic conditions

**Figure 3:**
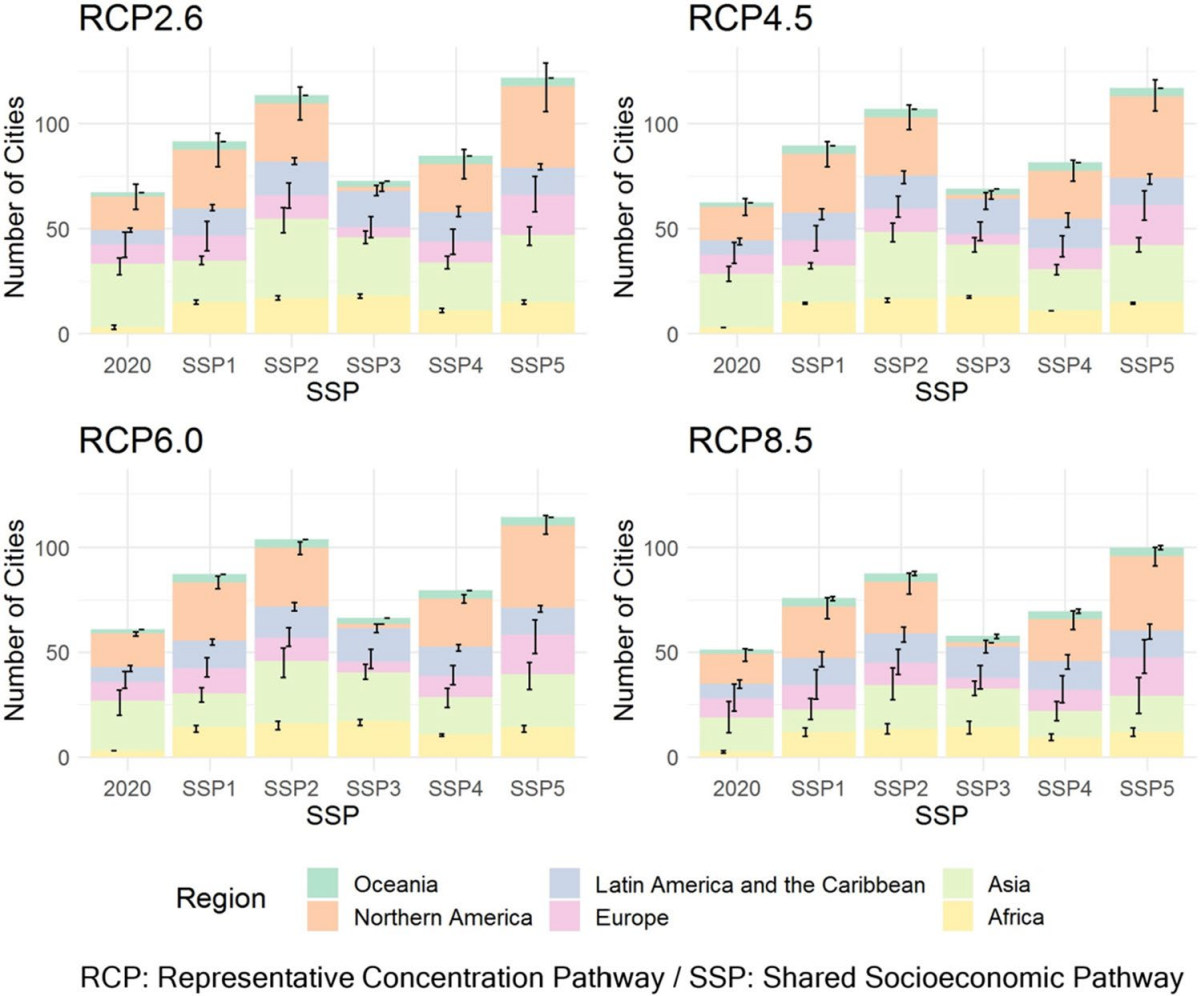
The number of cities that can host the Olympic marathon in August in the late-twenty-first century (2080–2099) by RCP/SSP/region. The bars represent the average of the seven GCMs. Error bars indicate the range between the maximum and minimum values of the seven GCMs.

Next, we estimate the number of cities that can host the Olympic marathon in the late twenty-first century (2080–2099), considering future socioeconomic scenarios (SSPs 1–5) (Fig. 3). Here, all combinations of the four RCPs and five SSPs are shown to identify the wide range of impacts due to climate change and socioeconomic conditions, but it should be noted that the combinations of RCP2.6 and SSP3 and RCP8.5 and SSPs 1–4 are infeasible^24^. The number of cities by country is shown in Supplementary Figs. 3–8, and the results for the mid-twenty-first century (2040–2059) are shown in Supplementary Figs. 9–14.

We selected target cities using the same criteria as in the evaluation of 70 cities for current socioeconomic conditions. The numbers of selected cities for the five SSPs are 94 for SSP1 (sustainability scenario), 120 for SSP2 (middle of the road scenario), 79 for SSP3 (regional rivalry scenario), 90 for SSP4 (inequality scenario), and 126 for SSP5 (fossil-fueled development scenario).

Globally, as in the results under current socioeconomic conditions, the number of cities that can host the Olympic marathon tends to decrease as the amount of greenhouse gas emissions increases, especially in Asia. The number of cities under each emission scenario is the highest for SSP5 and the lowest for SSP3. Under all SSPs, the number of possible host cities increases from the current socioeconomic conditions (2020). For example, under RCP2.6, the number of cities ranges from 72.7 (SSP3) to 122 (SSP5), while under RCP8.5, the number of cities ranges from 57.6 (SSP3) to 99.9 (SSP5).

For all SSPs, the number of possible cities under RCP8.5 is approximately 20% lower than that under RCP2.6, and the impact of the emission scenarios is apparent. Dozens of emerging cities, mainly in Asia, that could host the summer Olympics due to economic development under the low emission scenarios will not be able to host the Olympics under RCP8.5, the highest emission scenario.

### Effects of adaptation measures on climate change impacts under future socioeconomic conditions

**Figure 4:**
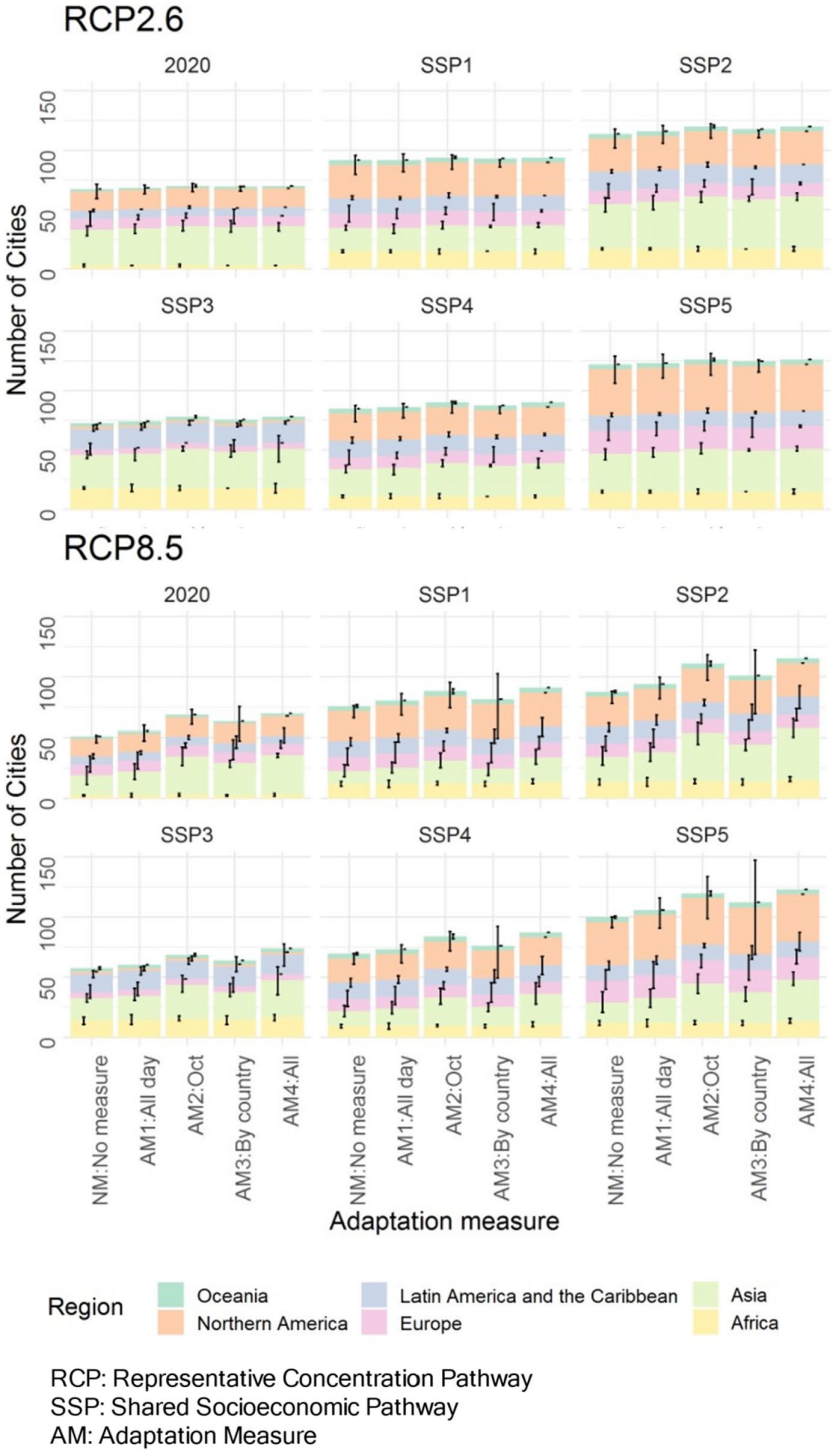
Change in the number of cities that can host the Olympic marathon due to AMs (RCP2.6, RCP8.5) in the late-twenty-first century (2080–2099) by SSP/region. The bars represent the average of the seven GCMs. Error bars indicate the range between the maximum and minimum values of the seven GCMs.

In this section, we evaluate the change in the number of cities that can host the Olympic marathon in August due to AMs in the late twenty-first century (2080–2099) (Fig. 4). The four types of AMs in Table 1 are adopted in this study. AM 1, 2, and 3 have been adopted or considered for the recent Games and can be reproduced with the spatiotemporal resolution of this study. AM 4 implements 1, 2, and 3 at the same time. The results for all four RCPs in the mid- and late-twenty-first centuries are shown in Supplementary Figs. 15–18.

**Table 1:** List of AMs adopted in this study with examples.

Adaptation measure 1 (AM1: All day)	Holding the Olympic marathon between 22:00 and 6:00 in August
Adaptation measure 2 (AM2: October)	Holding the Olympic marathon between 7:00 and 21:00 in October Example: Since 1980, the men's marathons at the 1988 Seoul Olympics and the 2000 Sydney Olympics were held in October^32^
Adaptation measure 3 (AM3: By country)	Holding the Olympic Games in multiple cities in a country and the Olympic marathon between 7:00 and 21:00 in August Example: The Tokyo Organizing Committee of the Olympic and Paralympic Games changed the venue for the women’s and men’s marathons and the race walk from Tokyo to Sapporo, according to International Olympic Committee (IOC) ’s strong recommendation concerning the excessive thermal load in Tokyo^34^
Adaptation measure 4 (AM4: All)	Implementing AMs 1 to 3 simultaneously. This measure has not been implemented or considered for the Olympic Games to the best of our knowledge
Cf. No measure	Holding the Olympic marathon between 7:00 and 21:00 in August. All Olympic marathons since the 1980 Moscow Olympics have been held during this time, except for the men's and women's marathons at the 1988 Seoul Olympics and 2000 Sydney Olympics, and the women's marathon at the 1996 Atlanta Olympics and 2020 Tokyo Olympics, which was moved up by an hour the day before to start at 6:00^28–32,35^

Under RCP2.6, the effects of AMs are limited due to the originally small decrease in the number of host cities (Fig. 4, upper part). Under RCP8.5, the effects of AMs are more pronounced, and the effects of single AMs are greater in the order of AM2, AM3, and AM1 (Fig. 4, lower part), while AM1 is the most common measure, and the majority of the possible cities’ increase occurs in Asia. The effect of AM4, in which all adaptation measures are implemented simultaneously, is the greatest, and more than 95% of the selected cities will be able to host the Olympic marathon even under RCP8.5.

**Figure 5:**
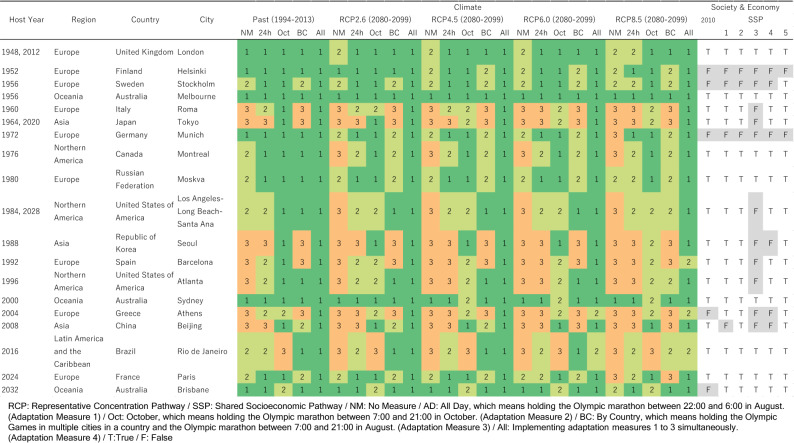
The WBGT levels in 19 cities that have hosted and/or are scheduled to host the Olympic Games (late-twenty-first century (2080–2099), RCPs 2.6–8.5, SSPs 1–5). The numbers in the columns under climate indicate the WBGT levels. In the columns under Society & Economy, “T” indicates that the city could be a candidate for hosting the Olympics under the socioeconomic conditions in 2010 or 2070–2090 (SSPs 1–5), while “F” indicates that the city is not a good candidate.

The WBGT levels under specific RCPs/SSPs/AMs in the late twenty-first century (2080–2099) are shown for 19 cities that have hosted and/or are scheduled to host the Games since 1948 (Fig. 5). Note that we excluded Mexico City, which was the host city in 1968 because its elevation of more than 1600 m did not meet our conditions for city selection.

If no adaptation measures are taken (NM), the number of cities with a high heat risk (WBGT level 3) will increase from 7 under the past climate to 10 to 12 (out of 19 cities) under the future climate. Even for Tokyo, which is assumed to be WBGT level 3 at the time of the 2020 Tokyo Olympics, the venues for marathons and race walks were relocated to Sapporo, a city more than 800 km away in the north, due to concerns about heat^36^. Thus, it is highly likely that equivalent measures will be required in other future host cities if they are assessed as WBGT level 3.

Among the single AMs, the effect of holding the event in October (AM2: Oct) is the highest. In most cities, the WBGT level will be 1 or 2 even under RCP8.5. On the other hand, in Rio de Janeiro, Sydney, and Brisbane, which are in the Southern Hemisphere, there are no effects of the measure, or rather, the WBGT level increases. Holding the event in multiple cities in a country (AM3: BC) may or may not be effective depending on the cities. Countries with more diverse domestic climates (cooler metropolises) may be advantageous. Notably, this study does not include cities for which sufficient climate data have not been collected, and the actual effect may be greater. The case where all AMs are implemented at the same time (AM4: All) is the most effective, with all cities under RCP2.6 and 4.5 and 17/16 cities under RCP6.0/8.5 having a WBGT level of 1. See the SI for the results for the mid-twenty-first century (Supplementary Fig. 19) and for all 165 cities in the mid- and late-twenty-first centuries (Supplementary Figs. 20–31).

## Discussion

The main contribution of this study is that it more comprehensively explains the feasibility of hosting the Olympic marathon in the future than previous studies^7,11^, considering the variations in climate change projections, the hourly climatic characteristics of cities, future socioeconomic conditions, and the effect of adaptation measures. As a result, the number of the 70 cities that can host the Olympic marathon under the socioeconomic conditions in 2020 decrease by approximately 27% globally under RCP8.5 in the late twenty-first century, with a particularly large decrease seen in Asia. When compared with the results of Smith et al.^11^, who assessed the situation as of 2085 under RCP8.5, the decrease in the number of potential host cities is more moderate in this study. Considering that the WBGT varies significantly within a day (see Supplementary Fig. 32 for an example), the fact that the evaluation is based on hourly WBGT rather than daily average WBGT, as in Smith et al.^11^, may have contributed to the difference in results. The use of seven GCMs in this study is also likely to contribute to the difference in results from Smith et al.^11^, who used two GCMs, since the error bars in the figures show significant variations among GCMs. Furthermore, we quantified the impacts of socioeconomic conditions (SSPs 1–5) and the effectiveness of adaptation measures in addition to the impacts of climate change.

The number of countries participating in the Games has increased from 14 in the first modern Games in Athens in 1896^37^ to 206 in Rio de Janeiro in 2016^38^, and since the Munich Olympics in 1972, each of the Games has been held on a different continent from the previous one, which suggests that regional diversity is becoming increasingly important in the Games. If the Games are to be held in various regions of the world in the future, adaptation measures including the four presented in this paper, are worth considering, in addition to mitigating the impacts of climate change by reducing greenhouse gas emissions. In particular, these adaptation measures will be necessary for many cities to be able to host marathons with relatively low heat risk (WBGT levels 1–2).

Hosting the Olympics in October (AM2) was found to be an effective adaptation measure, but it requires negotiation with broadcasters in North America (US and Canada), which pays the majority of the International Olympic Committee's broadcasting rights fees^39^. In North America, July and August are off-season for sports, which may be the reason why most of the recent Games have been held in those months^40^. The share of broadcasting rights fees from Asia, Europe, Central America, South America, and the Caribbean is increasing^39^, but it is not clear whether they will be sufficient to offset the possible decline in broadcasting rights fees for North America. In addition, another effective measure for holding the Games in multiple cities in a country (AM3) may increase the cost related to infrastructure, security, and logistics; therefore, careful consideration of cost-effectiveness is necessary.

For example, holding the Games in a city with existing infrastructure and experience in hosting large-scale sporting events and/or limiting the number of spectators and people involved in the Games would reduce the costs associated with AM2 and AM3^41,42^. The above proposal is consistent with the proposal by Muller et al.^10^ from the viewpoint of the sustainability of the Games (e.g., drastic reduction in the scale of the Games or rotating the Games among the existing host cities).

Some issues should be considered in the future. (1) The Games include many outdoor events other than the marathon. It is desirable to evaluate the thermal conditions for such events and to consider possible competition programs within 16 days, which is the basic duration of the Games^43^. (2) Although the focus of this study is on athletes, the heat risk to spectators, who make up the majority of those involved, should be considered as well. (3) In this study, the evaluation was based on the WBGT of representative points in each city, and therefore, microclimatic conditions in the city and adaptation measures, such as the construction of heat-shielding pavements, planting of trees, and misting, were not considered. Such evaluations require a large computational resource for modeling and/or detailed measurements in the field; therefore, it is realistic to use these methods for a smaller number of cities. (4) The cities selected in this study may differ from those that can host the Olympic Games in the future due to the lack of relevant data. Factors such as political rights, experience in hosting world championships, the population support for the Games, dispute with the IOC, and existing stadium infrastructure are also important for being selected as a host city^44^, but it is difficult to predict the situation of such factors in the mid- and late twenty-first centuries. Therefore, we did not include them in the city selection. Additionally, many cities particularly in Africa and Latin America are excluded due to the lack of sufficient meteorological observation data to reproduce the climatic characteristics of cities, even though they have sufficient socioeconomic conditions to be selected. Moreover, from the perspective of sustainability, Olympic Agenda 2020^45^, presented by the IOC as a reform plan for the Olympic Movement, encourages the use of simple temporary facilities rather than new venues, and it allows the games to be held in locations other than the host city (e.g., where facilities already exist). Therefore, it is possible that smaller cities than those targeted in this study will be allowed to host the Olympics in the future. Based on the above, it is necessary to review the conditions for selecting cities in the future, taking into account the improvement in related data and the trend of sustainability improvement in the Olympics. (5) The WBGT, which was used as the index in this study, has several limitations, including the tendency to underestimate heat in environments where sweat evaporation is limited (high humidity and low wind speed), the inability to take into account conditions such as exercise intensity and clothing, and the lack of evidence on the relationship with health risks in sports^46,47^. In the future, it is necessary to develop more robust methods for assessing health risks in marathon running and sports in general.

## Methods

### Selection of cities for evaluation

Referring to the papers that studied the selection of host cities for the Games^44,48^ and the conditions of the historical host cities, we selected 165 cities in 66 countries based on the following four perspectives: (1) the urban population, (2) national GDP and its growth rates, (3) elevation, and (4) the availability of meteorological observation data to reproduce the climatic characteristics of the city. Regarding socioeconomic conditions, (1) and (2), since the selection of host cities generally starts approximately 10 years before the Games^48^, this study use data from 2010 (for 2020), the 2030–2050 period (for 2040–2059), and the 2070–2090 period (for 2080–2099), 10 years before the evaluation period. The specific conditions for the four perspectives are described as follows.

Condition 1: The city has a population of at least 2.5 million. A study of bids for the 1992–2020 Summer Olympic Games pointed out that a city population of 2.5 million or more is an important factor in being selected as a host city^44^. In reality, competitions are rarely all held in a single city, and in most cases, competitions are also held in neighboring cities. Therefore, in this study, the selection is made based on the population of urban agglomerations (“the population contained within the contours of a contiguous territory inhabited at urban density levels without regard for administrative boundaries”^21^). We use data from 2010 (for 2020), the 2030–2050 period (for 2040–2059), and the 2070–2090 period (for 2080–2099). The data for each period is acquired or calculated using the following methods.

2010: The annual population of urban agglomerations in 2010^21^.

2030–2050: These data were calculated by multiplying the urban population in 2020 by the ratio of the projected national population in 2020 and the average population from 2030 to 2050 in five-year intervals for each SSP^22^.

2070–2090: These data were calculated by multiplying the urban population in 2020 by the ratio of the projected national population in 2020 and the average population from 2070 to 2090 in five-year intervals for each SSP^22^.

Condition 2: The national GDP is no less than $300 billion (PPP, Int'l $ 2005) and the GDP growth rate is above 0%. Olympic-related expenditures are often made on a national basis and on a city basis. After the 1992 Summer Olympic Games in Barcelona, no host city had a national GDP of less than $300 billion 10 years before the Games^49^. Therefore, we set the threshold at $300 billion. Regarding GDP, its medium-term growth rate is important for being selected as a host city^44^, and after the 1992 Summer Olympics in Barcelona, there was no host city with an average national GDP growth rate of less than 0% from 15 to 10 years before the Games. For these reasons, we set a GDP growth rate of above 0% as a condition. We use data from 2010 (GDP for 2020), the 2010–2015 period (GDP growth rate for 2020), the 2030–2050 period (GDP and its growth rate for 2040–2059), and the 2070–2090 period (GDP and its growth rate for 2080–2099)^22^. For each SSP, the national GDPs in 2010, the average GDP from 2030 to 2050, and the average GDP from 2070 to 2090 are used^22^.

Condition 3: The elevation of the city is less than 1,600 m^11^. Given the negative effects of a high elevation on long-distance running, including marathons that became apparent at the 1968 Mexico City Olympics^9^, cities with elevations higher than 1,600 m should not be viable for hosting marathon competitions.

Condition 4: There is a meteorological station within 20 km of the representative point of the city, with at least 2,500 data points collected from May to October (summer season in the Northern Hemisphere) over multiple years at intervals of 3 h or less. This condition was established to adequately reproduce the site-specific WBGT, referring to the SI of Takakura et al.^20^. We used terrestrial weather station data^51^ and solar radiation data^52^ corresponding to the weather stations.

### Calculating site-specific hourly resolution WBGT

To consider holding a marathon in a particular city for a few hours of the day, it is necessary to calculate the site-specific hourly resolution WBGT. The environmental conditions during Olympic competitions vary even from start to finish during the same race^9^. In many cases, the output of GCMs does not provide diurnal variations, and the spatial resolution is coarse (e.g., horizontal resolution > 50 km), so it is not suitable for this study. Dynamical downscaling of GCM output using regional climate models (RCMs) is a common solution for spatiotemporally detailed calculations, but it is computationally expensive and not suitable for extensive global studies, such as this one. Therefore, we adopt the method of Takakura et al.^20^, which can provide site-specific hourly resolution WBGT prediction with only relatively simple calculations using GCM outputs, and we can achieve the objective of this study with a reasonable amount of computation.

In Takakura et al.^20^, bias correction of WBGT calculated from each grid of GCMs was performed using the observation records of WBGT in six cities in Japan. In this study, however, we use the hourly resolution meteorological data of each city to calculate the equivalent WBGT data using the method of Liljegren et al.^53^ since most of the cities in the world do not have published WBGT observation records. Additionally, we used natural spline interpolation to create 24-h data for cities where data existed at 3-h intervals or less and excluded other cities since many cities do not have a complete set of 24-h observation data. For more explanations of the calculation, see Takakura et al.^20^ and Supplementary Fig. 33.

### Statistical analysis

The feasibility of hosting the Olympic marathon in each city is assessed by comparing the 90th percentile of the WBGT between 7:00 and 21:00 in the RCP/target period (e.g. RCP4.5/2080–2099), averaged over seven GCMs, with the conditions of four WBGT levels defined in “Climate change impacts under current socioeconomic conditions”. To mitigate the effects of interannual variability of the climate, the period covered is 20 years, and this setting is commonly used in IPCC reports^1^. The 90th percentile is used to identify the 90% probability of the definition of WBGT levels. In Figs. 1, 2, 3 and 4, the maximum and minimum 90th percentiles of seven GCMs are shown as error bars to represent the variability among climate models.

### Setting of adaptation measures

We evaluate the effect of four AMs that have been adopted or considered in recent Games (except AM4) and can be reproduced with the spatiotemporal resolution of this study. However, it has not been confirmed whether these measures were considered heat countermeasures when they were adopted or considered in the past. See Table 1 for details.

## Supplementary Information

Supplementary Information.

## Data Availability

The datasets generated and/or analyzed during the current study are available from the corresponding author upon reasonable request.
