# Supplementary material for: Feasibility of the Olympic marathon under climatic and socioeconomic change
Source: Sci Rep. 2022 Mar 7;12:4010. doi: 10.1038/s41598-022-07934-6 (PMC8901618; doi:10.1038/s41598-022-07934-6)

Supplementary Information for “Feasibility of the Olympic marathon under climatic and socioeconomic change”

| 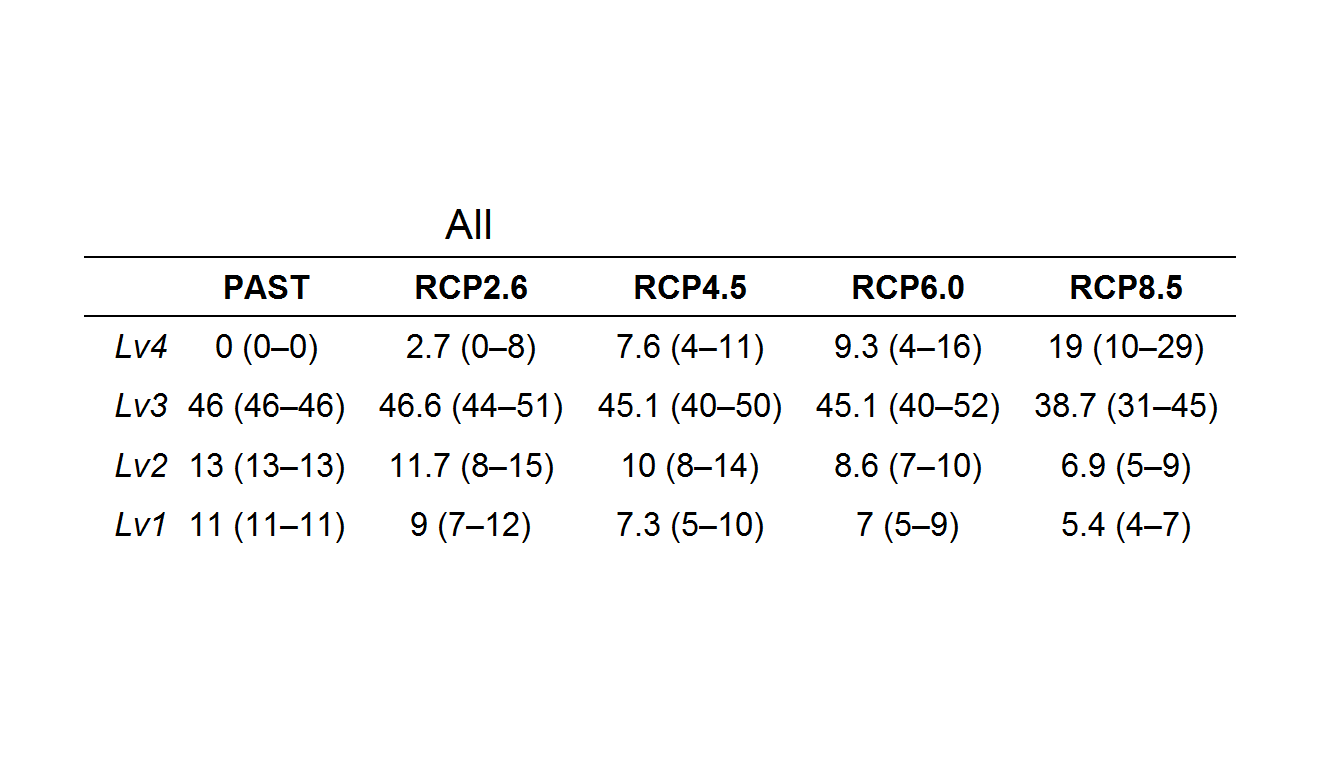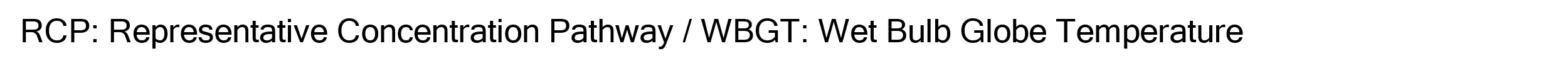 |
| --- |

Supplementary Table. 1 Number of cities by WBGT level in August in the late-21st century (2080–2099). The cities are selected under the socioeconomic conditions in 2010. The figures show the average values based on seven GCMs. The figures in parentheses represent the "minimum–maximum" values of the seven GCMs.

| 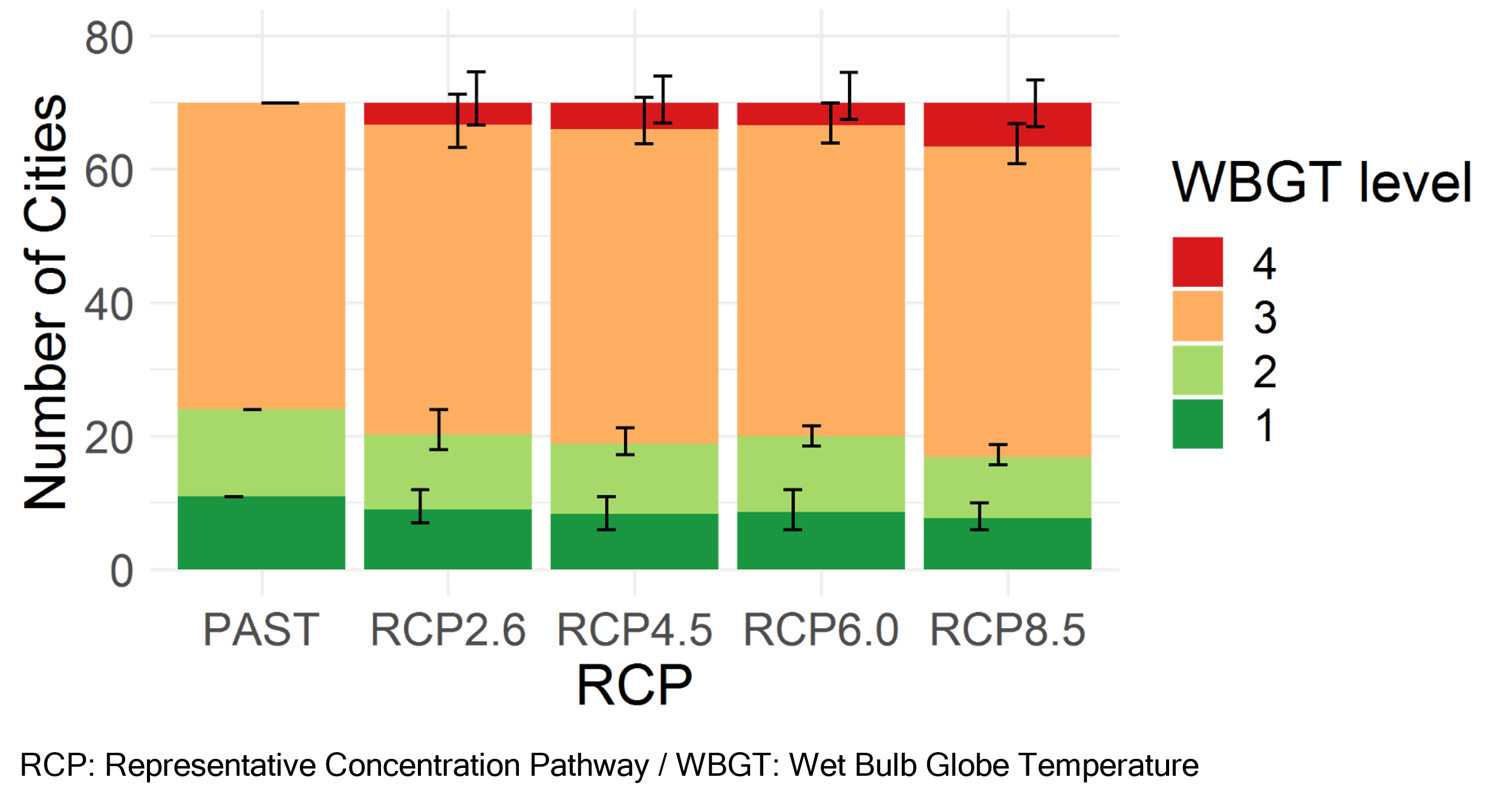 |
| --- |

1. WBGT levels of 70 cities in August in the mid-21st century (2040–2059). The cities are selected under the socioeconomic conditions in 2010. The bars represent the average of the seven GCMs. Error bars indicate the range between the maximum and minimum values of the seven GCMs.

| 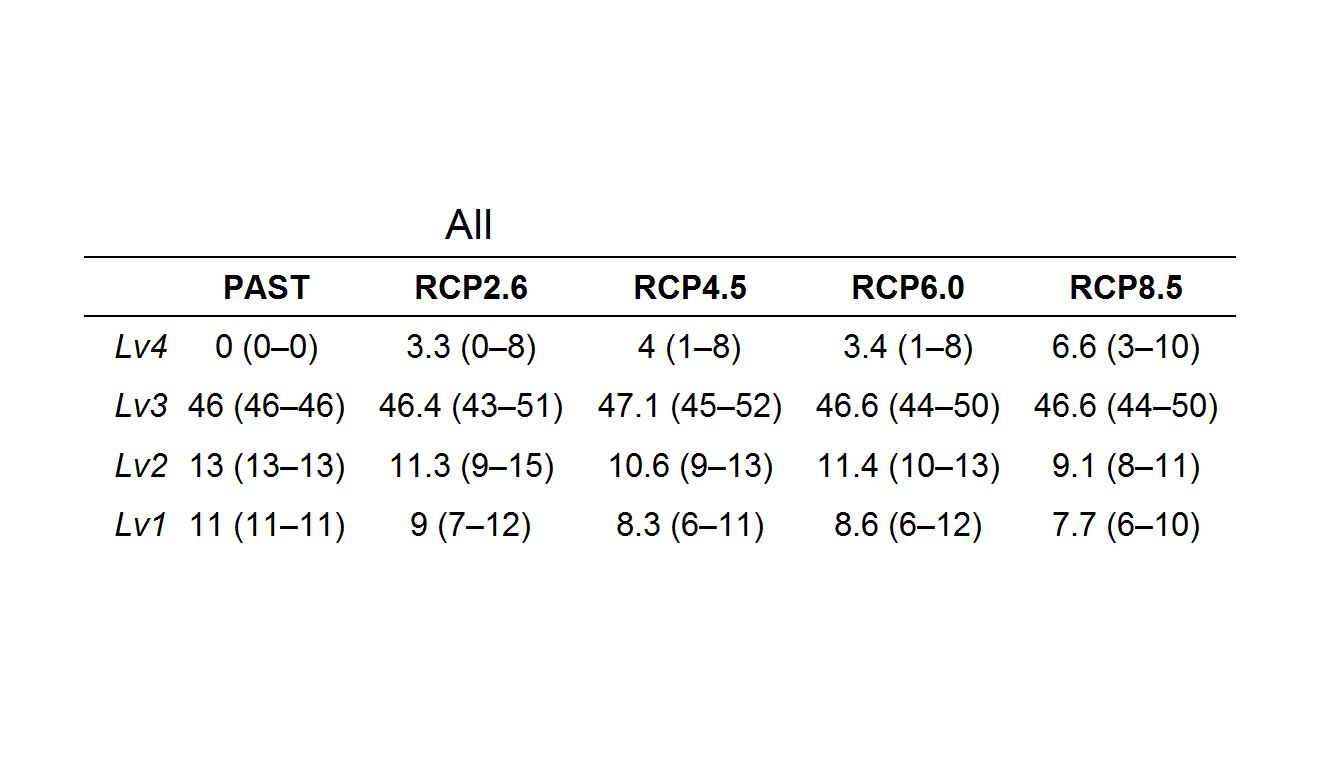  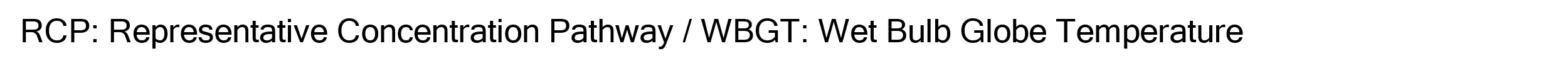 |
| --- |

Supplementary Table. 2 Number of cities by WBGT level in August in the mid-21^st^ century (2040–2059). The cities are selected under the socioeconomic conditions in 2010. The figures show the average values based on seven GCMs. The figures in parentheses represent the “minimum–­­m­­aximum” values of the seven GCMs.

|  |
| --- |

1. WBGT levels of 70 cities in August of the mid-21st century (2040–2059) by region. The cities are selected under the socioeconomic conditions in 2010. The bars represent the average of the seven GCMs. Error bars indicate the range between the maximum and minimum values of the seven GCMs.

| 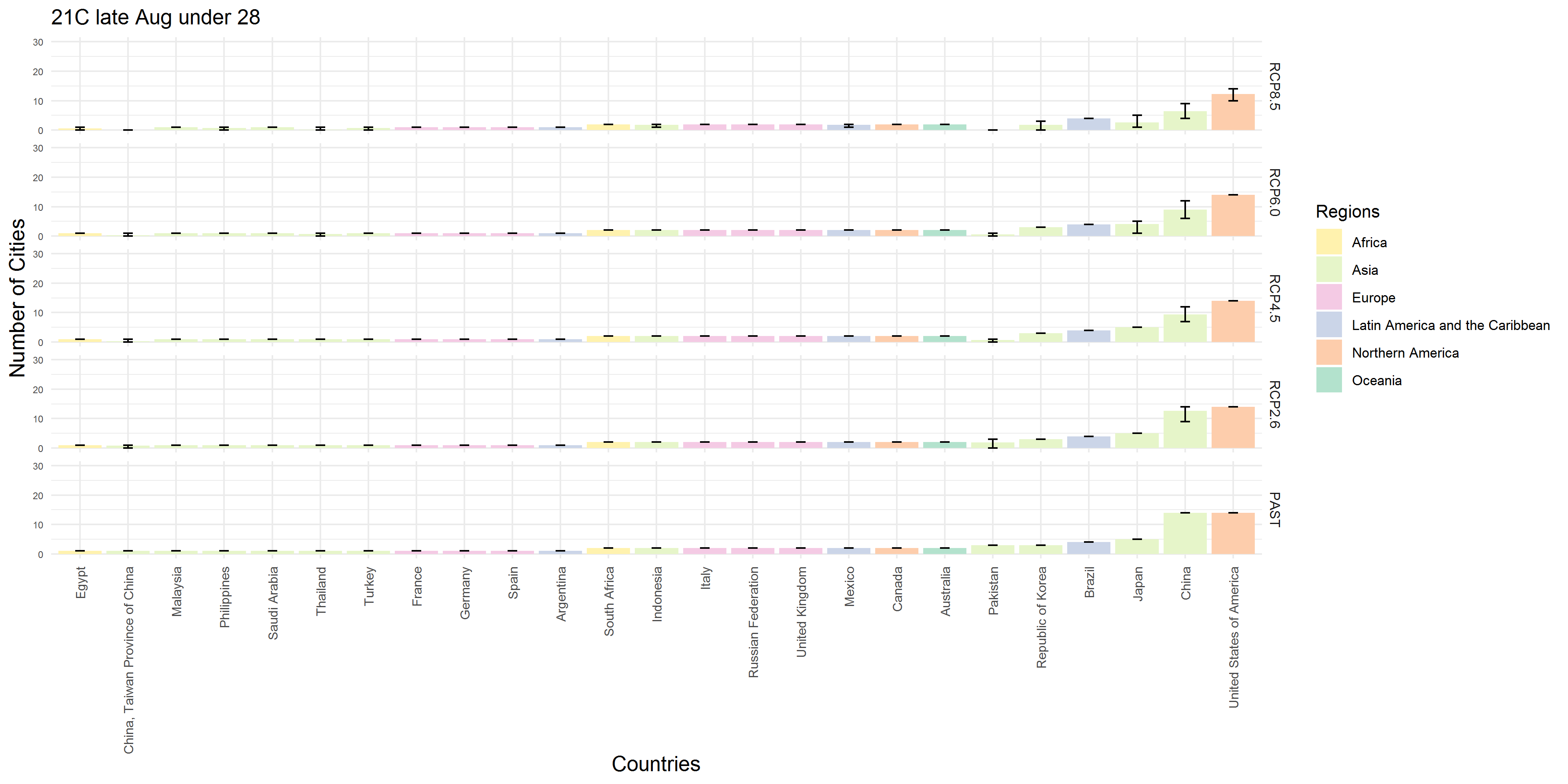  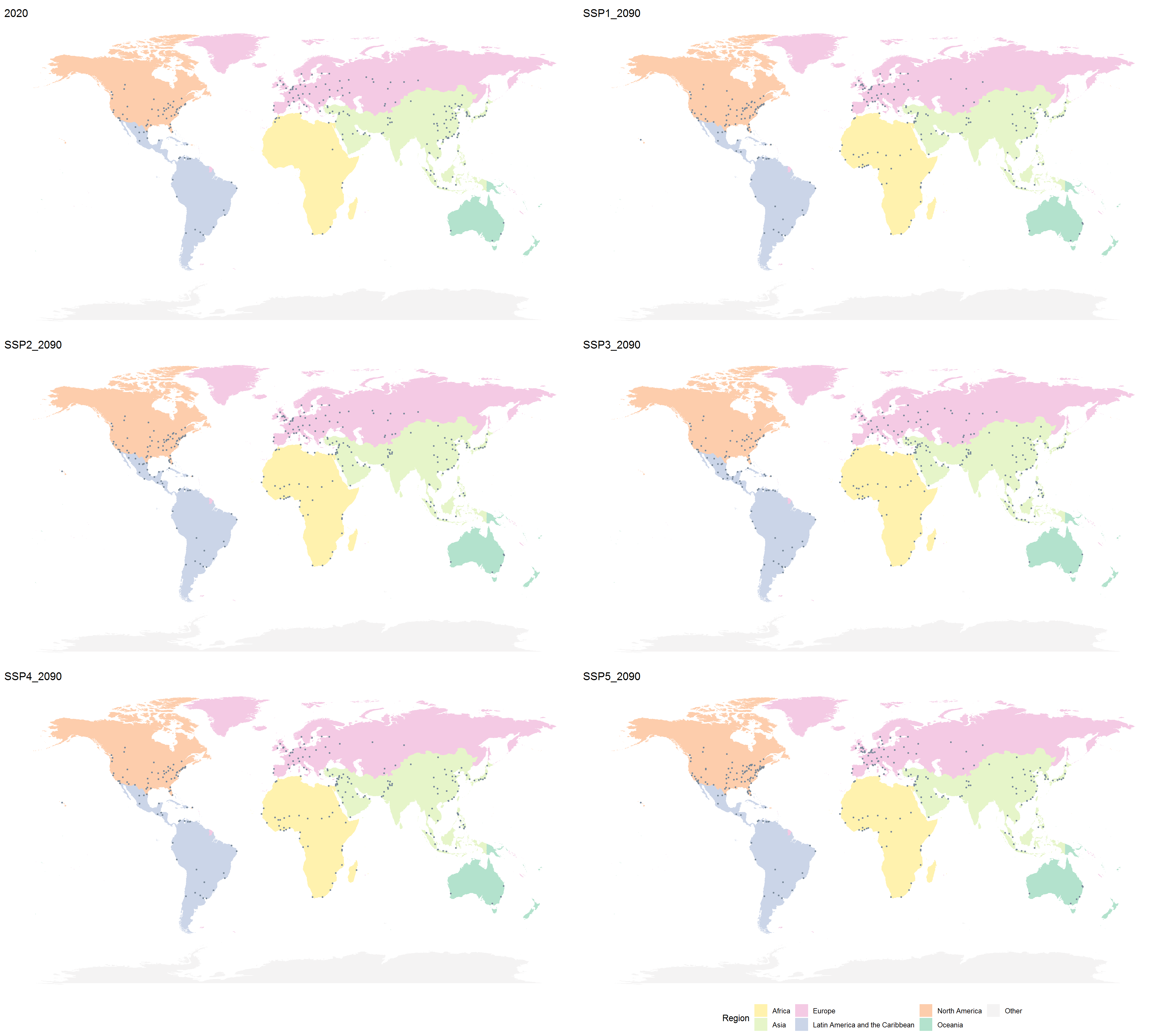  RCP: Representative Concentration Pathway |
| --- |

1. The number of cities that can host the Olympic marathon (WBGT levels 1 to 3) in the late-21st century (2080–2099) under current social-economic conditions by RCP/country. Note that the results are only for the cities covered in this study, and the distribution of the number of cities by country may be different if cities that do not publish meteorological data in the NOAA database are included. Error bars indicate the range between the maximum and minimum values of the seven GCMs.

| 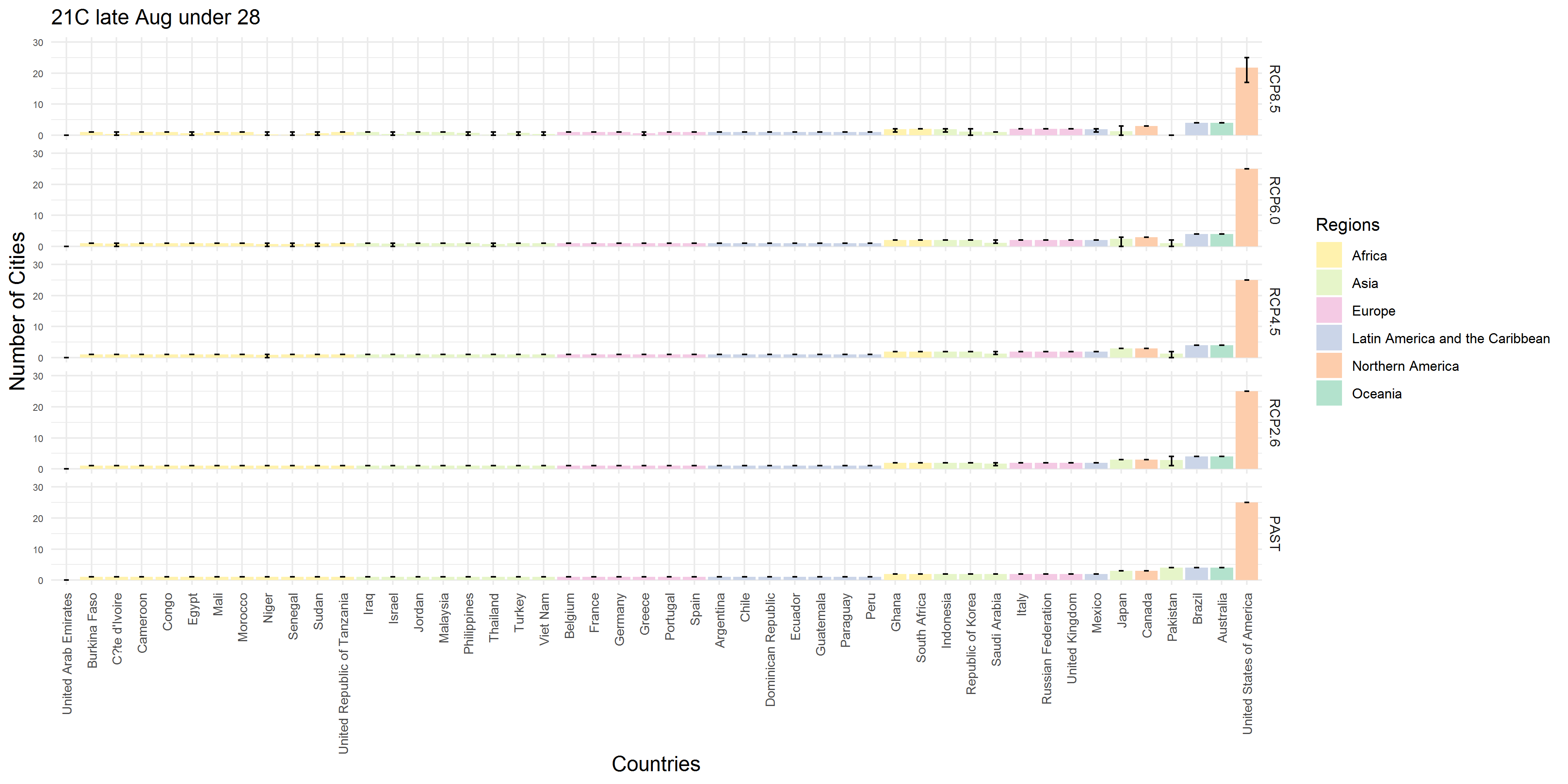  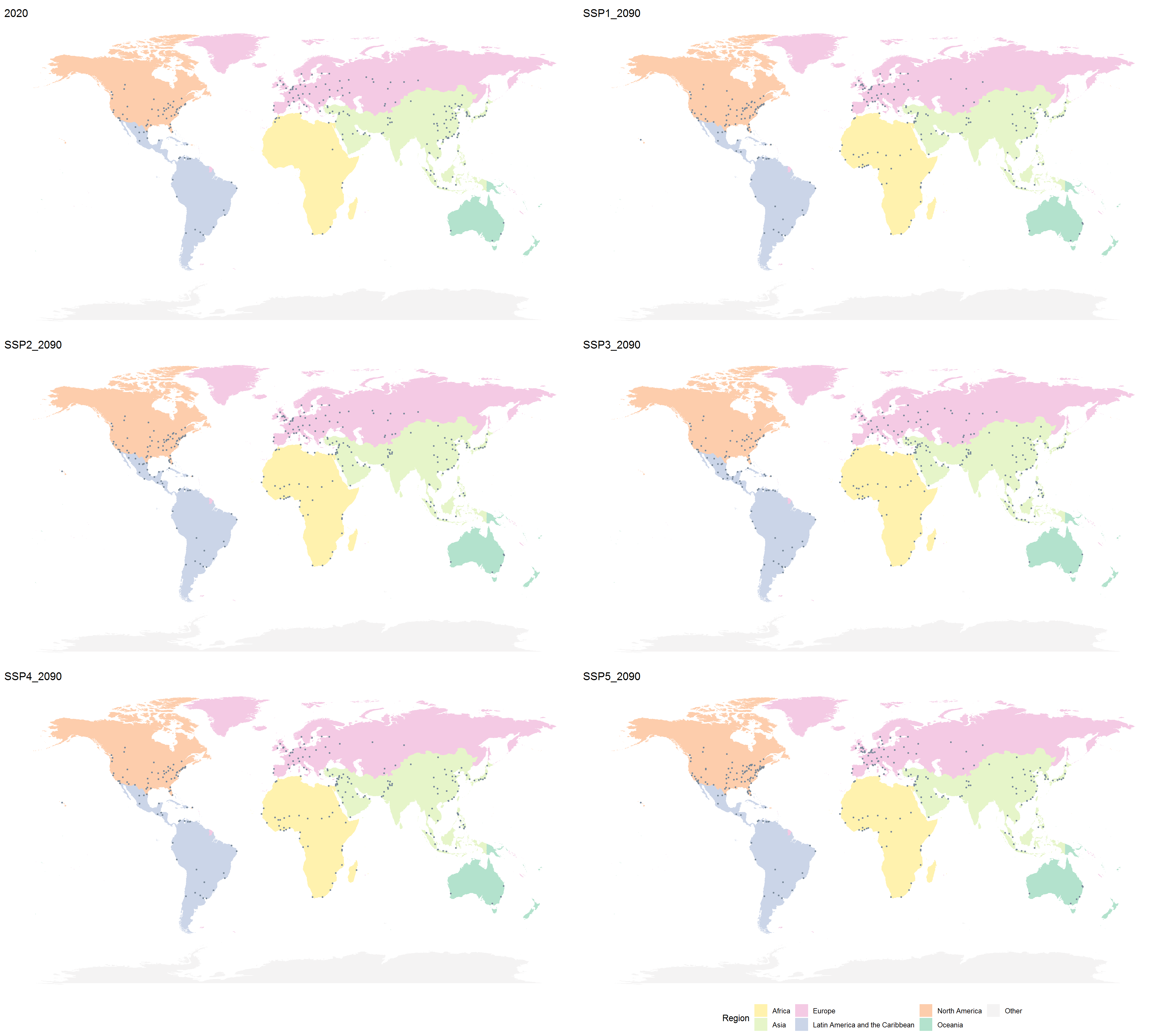  RCP: Representative Concentration Pathway |
| --- |

1. The number of cities that can host the Olympic marathon (WBGT levels 1 to 3) in the late-21st century (2080–2099) under SSP1 by RCP/country. Note that the results are only for the cities covered in this study, and the distribution of the number of cities by country may be different if cities that do not publish meteorological data in the NOAA database are included. Error bars indicate the range between the maximum and minimum values of the seven GCMs.

| 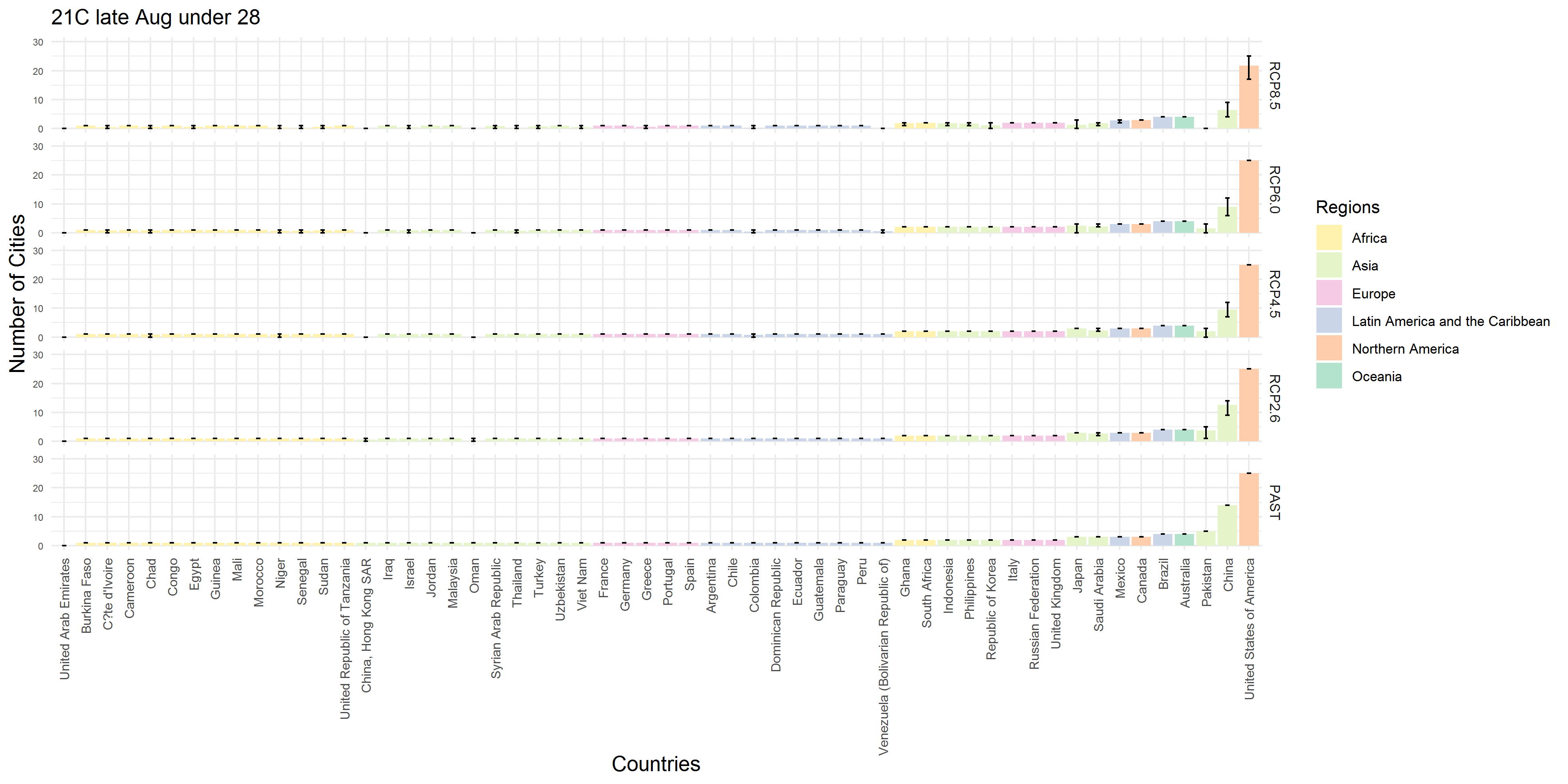  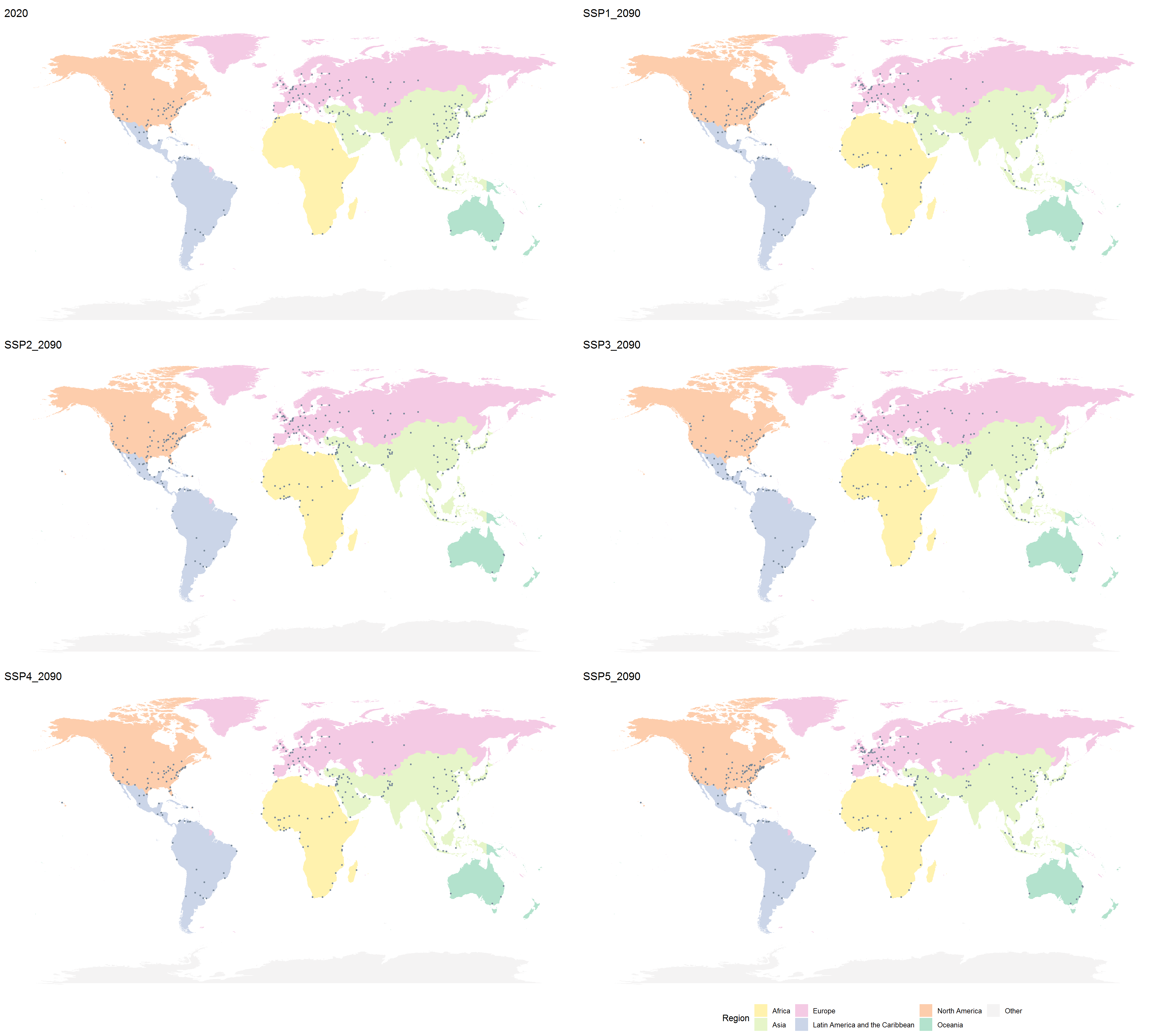  RCP: Representative Concentration Pathway |
| --- |

1. The number of cities that can host the Olympic marathon (WBGT levels 1 to 3) in the late-21st century (2080–2099) under SSP2 by RCP/country. Note that the results are only for the cities covered in this study, and the distribution of the number of cities by country may be different if cities that do not publish meteorological data in the NOAA database are included. Error bars indicate the range between the maximum and minimum values of the seven GCMs.

| 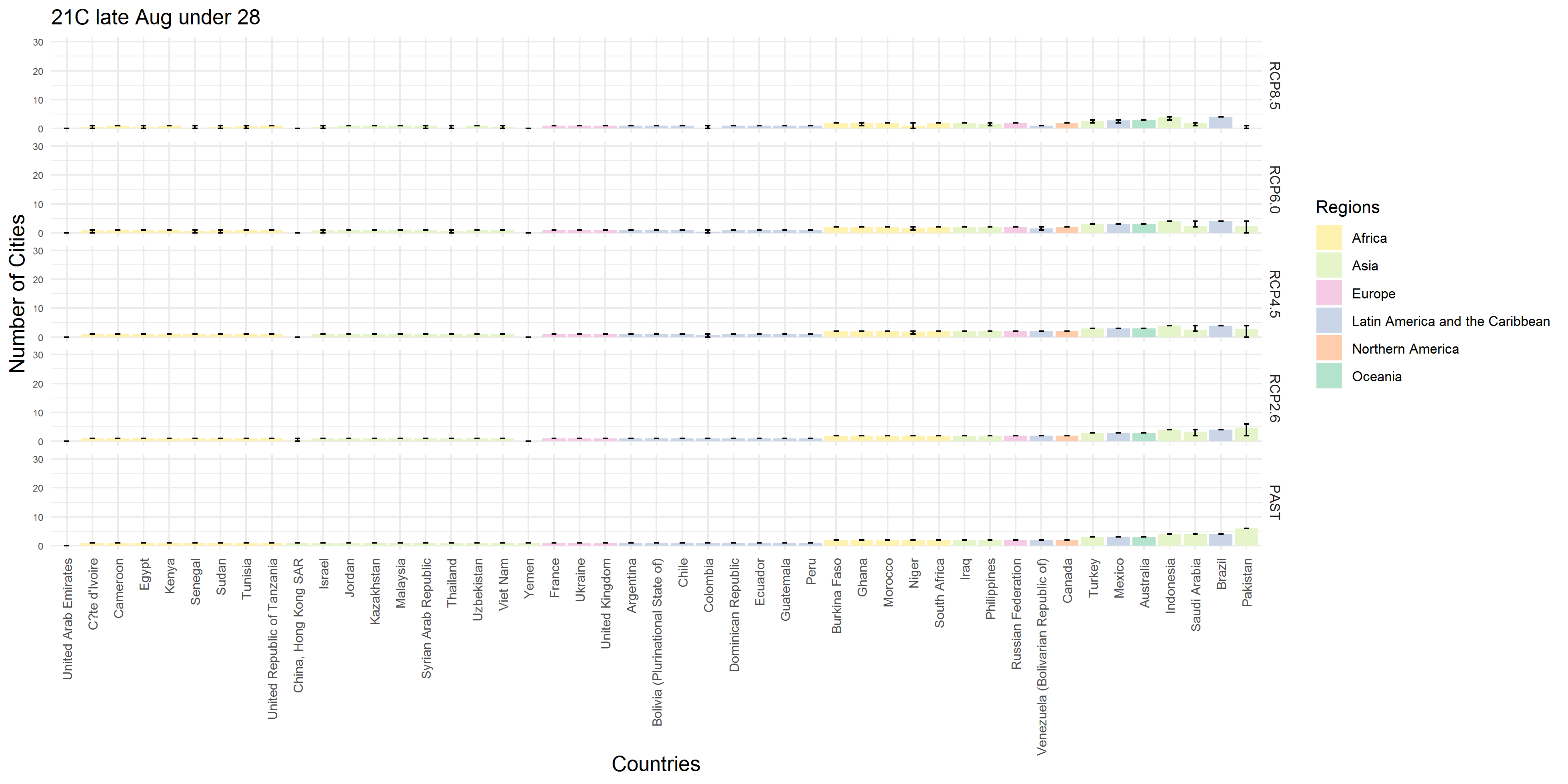  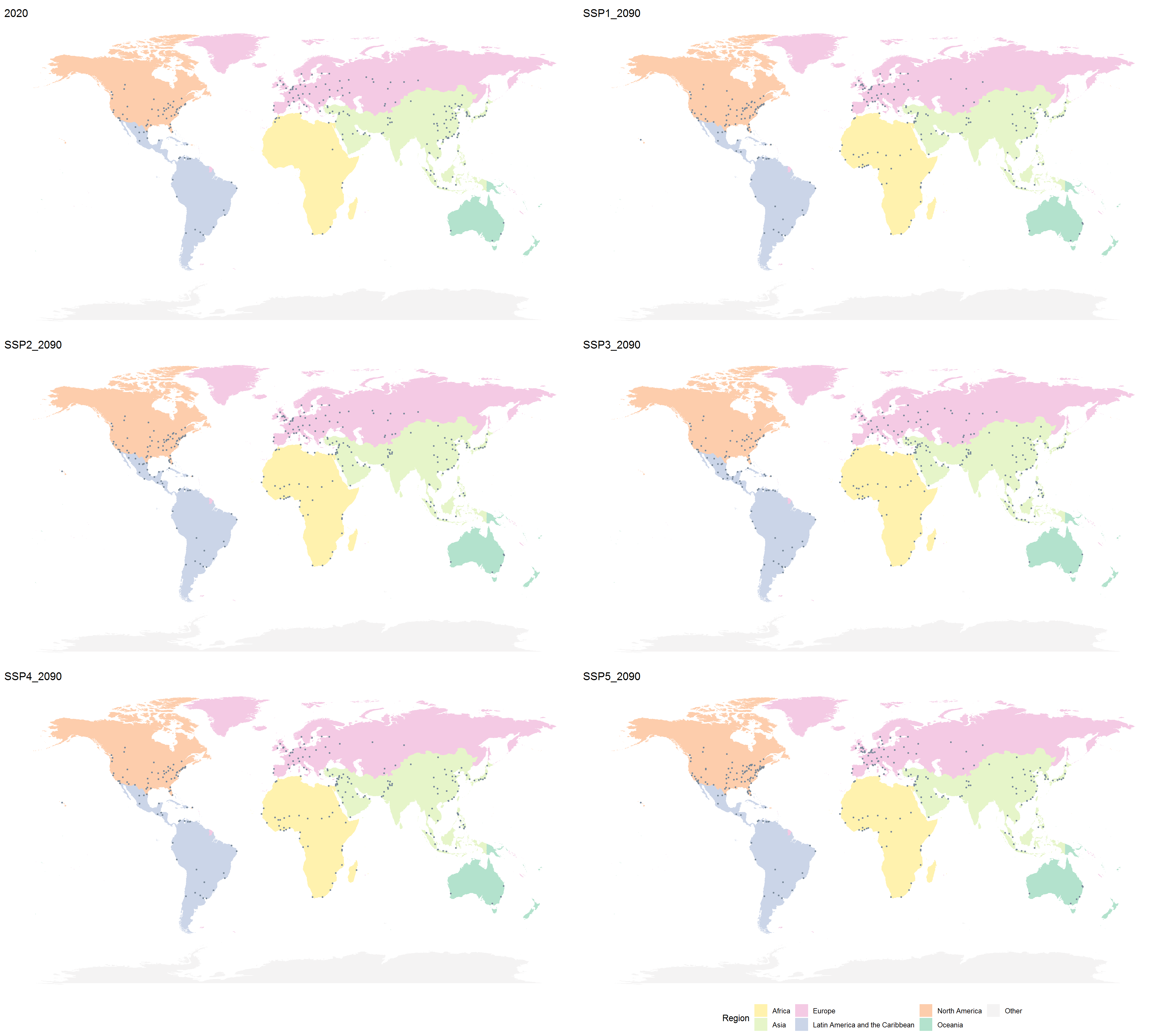  RCP: Representative Concentration Pathway |
| --- |

1. The number of cities that can host the Olympic marathon (WBGT levels 1 to 3) in the late-21st century (2080–2099) under SSP3 by RCP/country. Note that the results are only for the cities covered in this study, and the distribution of the number of cities by country may be different if cities that do not publish meteorological data in the NOAA database are included. Error bars indicate the range between the maximum and minimum values of the seven GCMs.

| 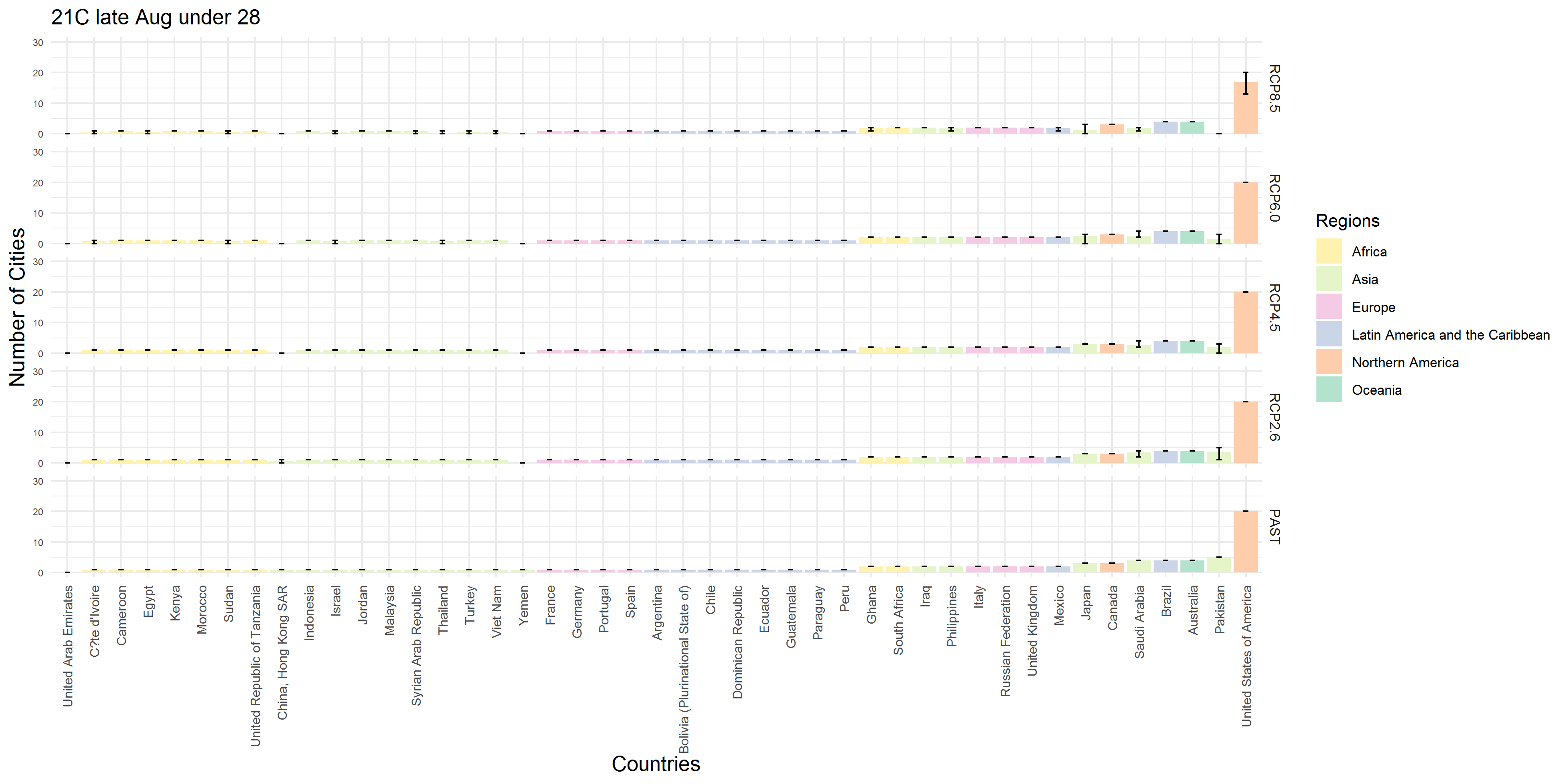  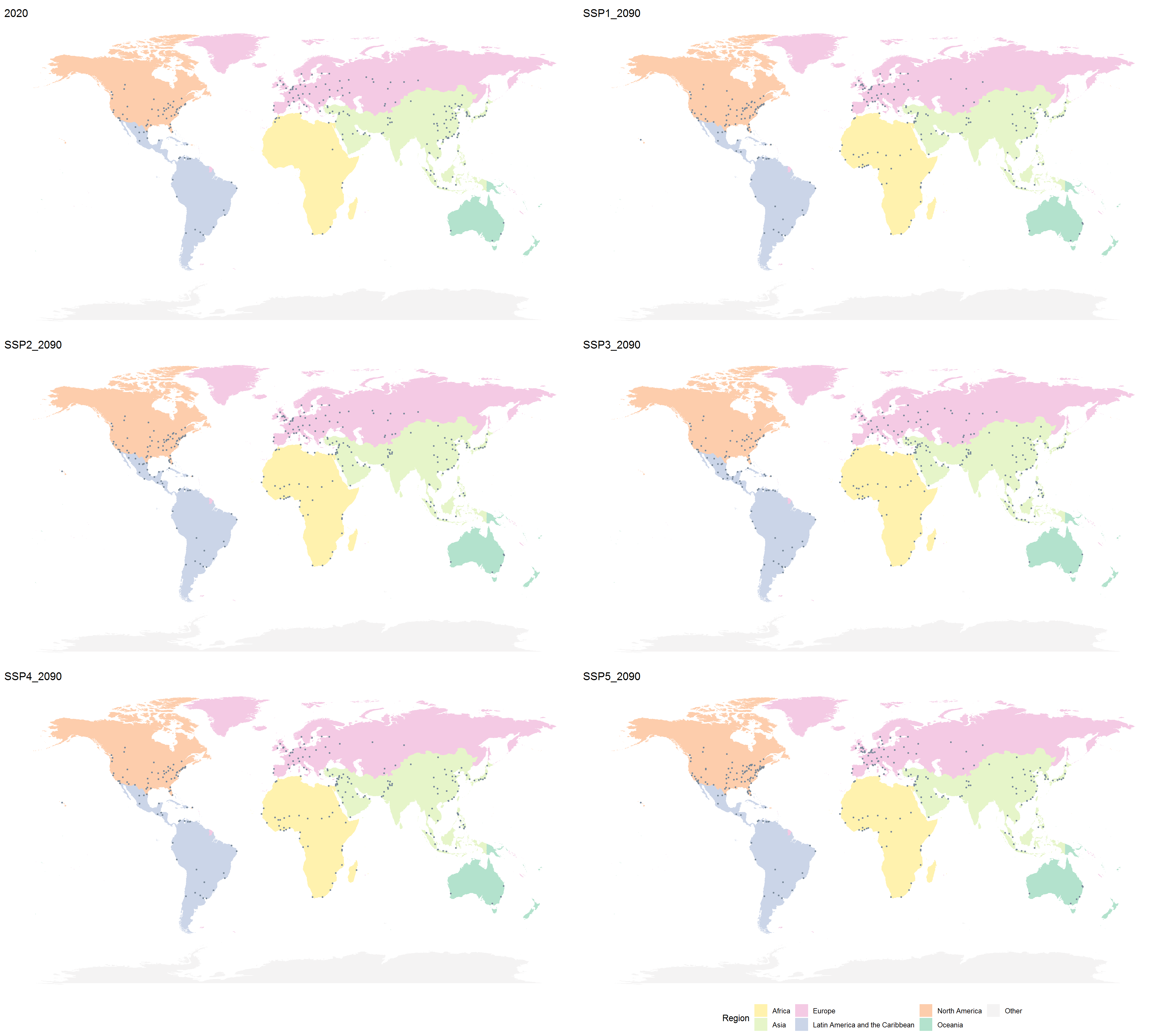  RCP: Representative Concentration Pathway |
| --- |

1. The number of cities that can host the Olympic marathon (WBGT levels 1 to 3) in the late-21st century (2080–2099) under SSP4 by RCP/country. Note that the results are only for the cities covered in this study, and the distribution of the number of cities by country may be different if cities that do not publish meteorological data in the NOAA database are included. Error bars indicate the range between the maximum and minimum values of the seven GCMs.

| 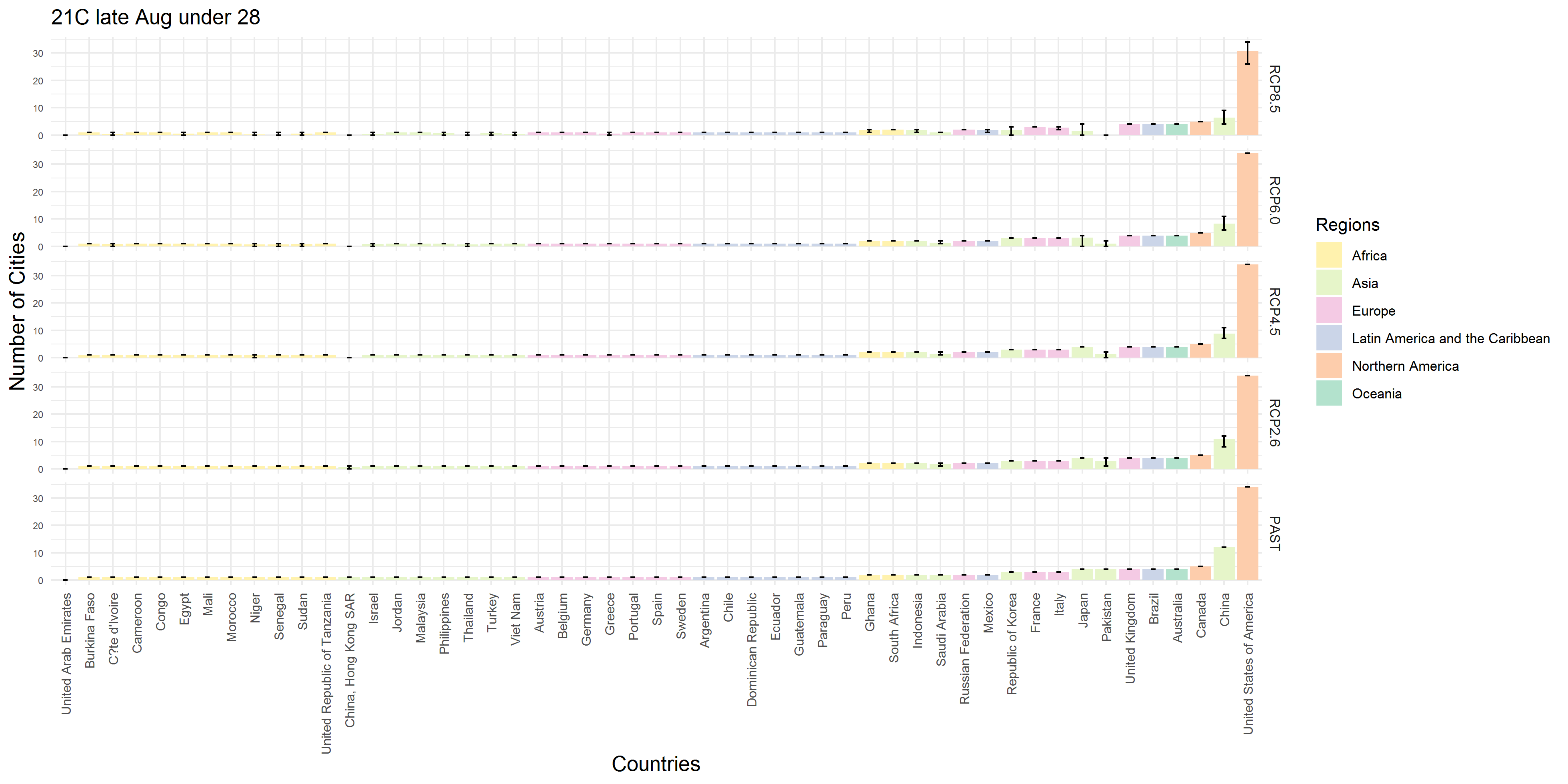  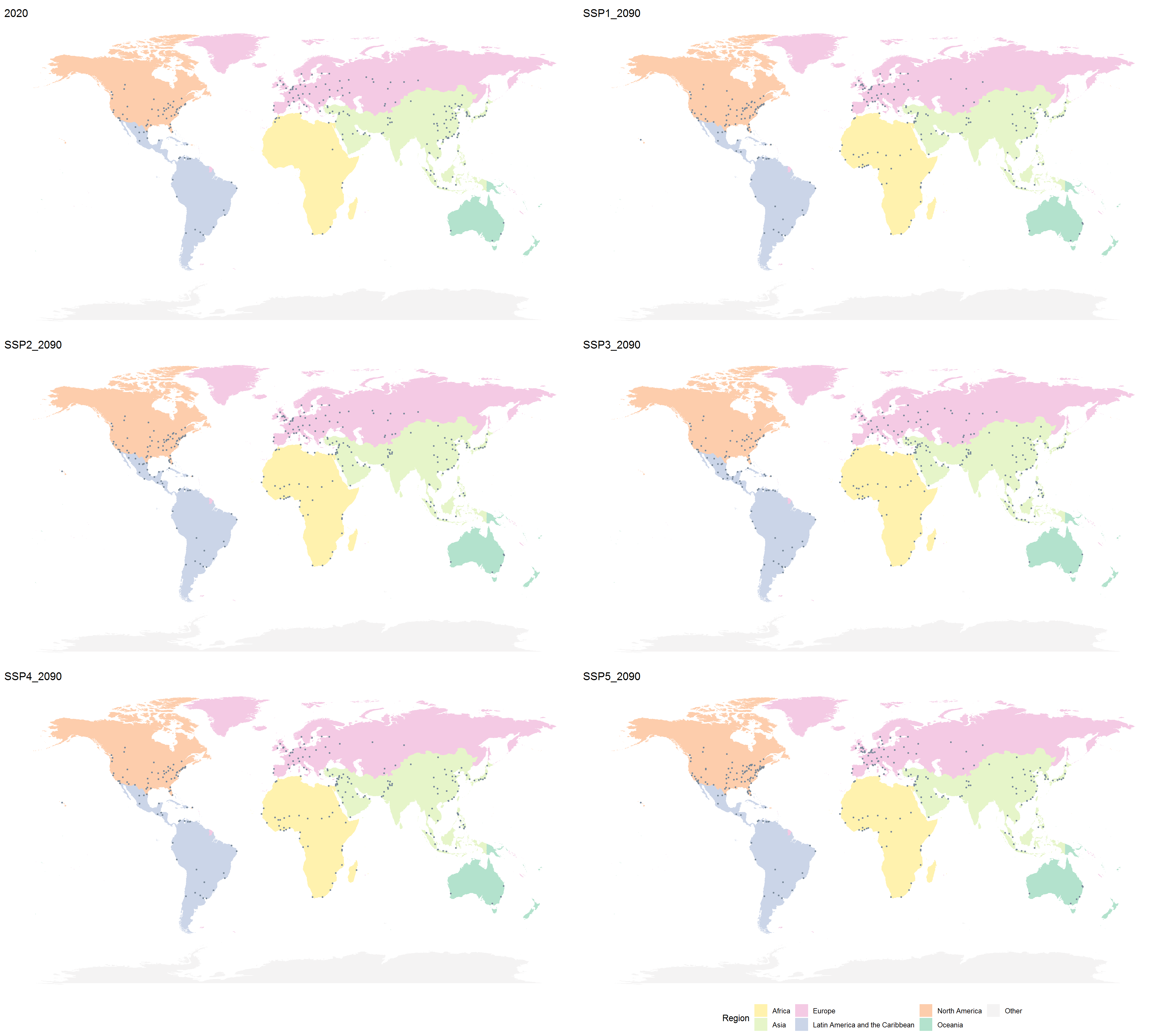  RCP: Representative Concentration Pathway |
| --- |

1. The number of cities that can host the Olympic marathon (WBGT levels 1 to 3) in the late-21st century (2080–2099) under SSP5 by RCP/country. Note that the results are only for the cities covered in this study, and the distribution of the number of cities by country may be different if cities that do not publish meteorological data in the NOAA database are included. Error bars indicate the range between the maximum and minimum values of the seven GCMs.

| 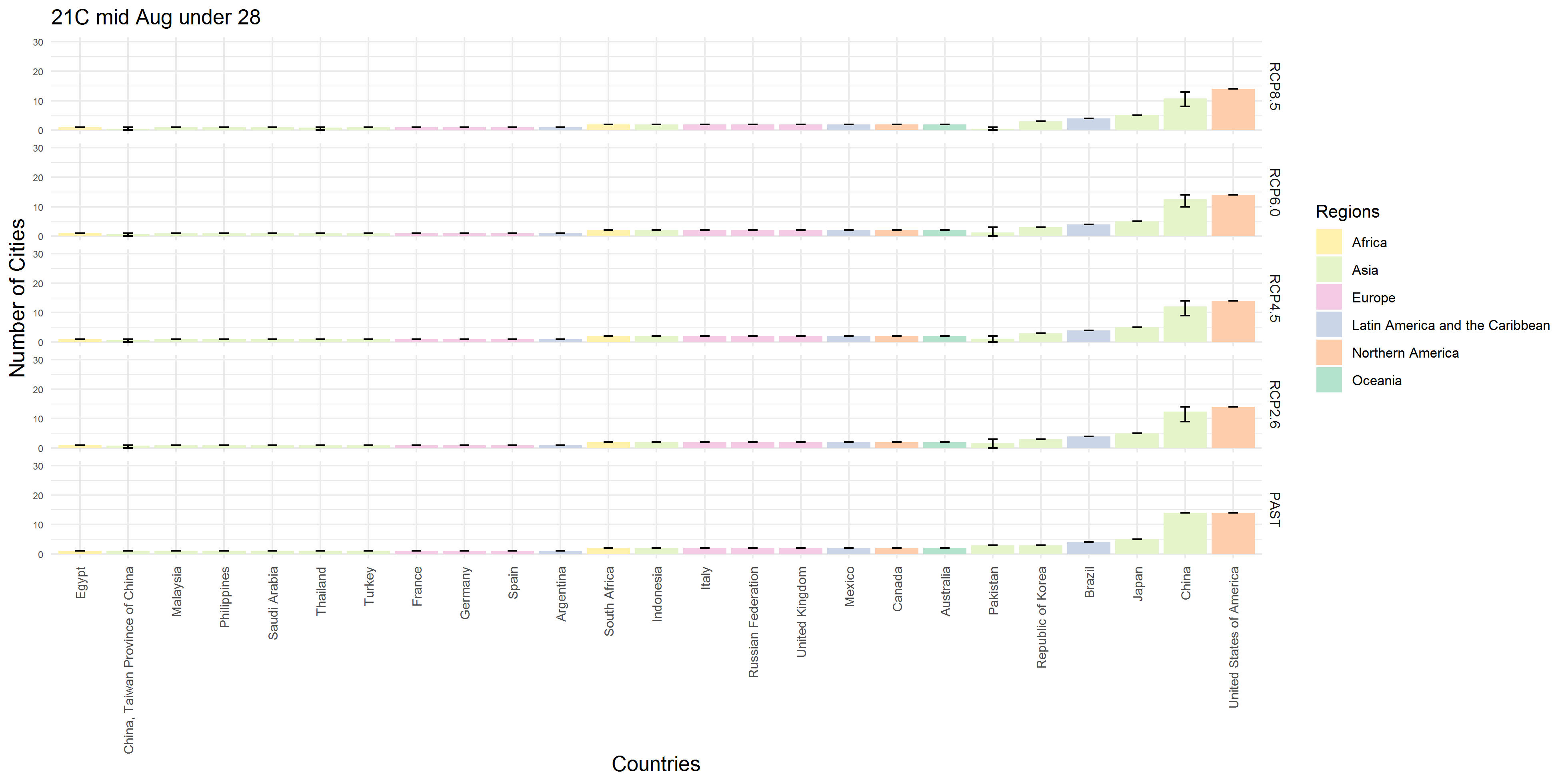  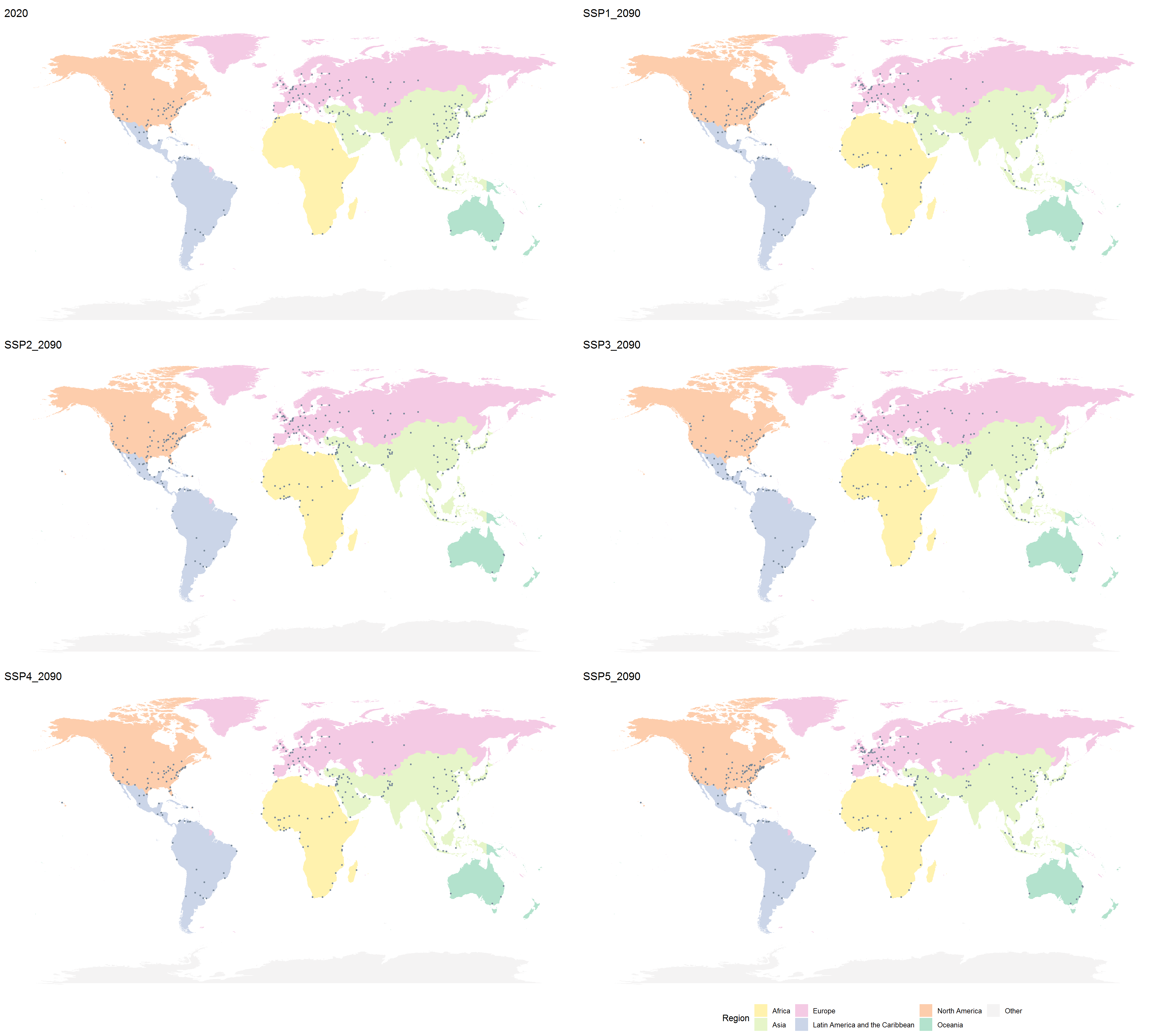  RCP: Representative Concentration Pathway |
| --- |

1. The number of cities that can host the Olympic marathon (WBGT levels 1 to 3) in the mid-21st century (2040–2059) under current social-economic conditions by RCP/country. Note that the results are only for the cities covered in this study, and the distribution of the number of cities by country may be different if cities that do not publish meteorological data in the NOAA database are included. Error bars indicate the range between the maximum and minimum values of the seven GCMs.

| 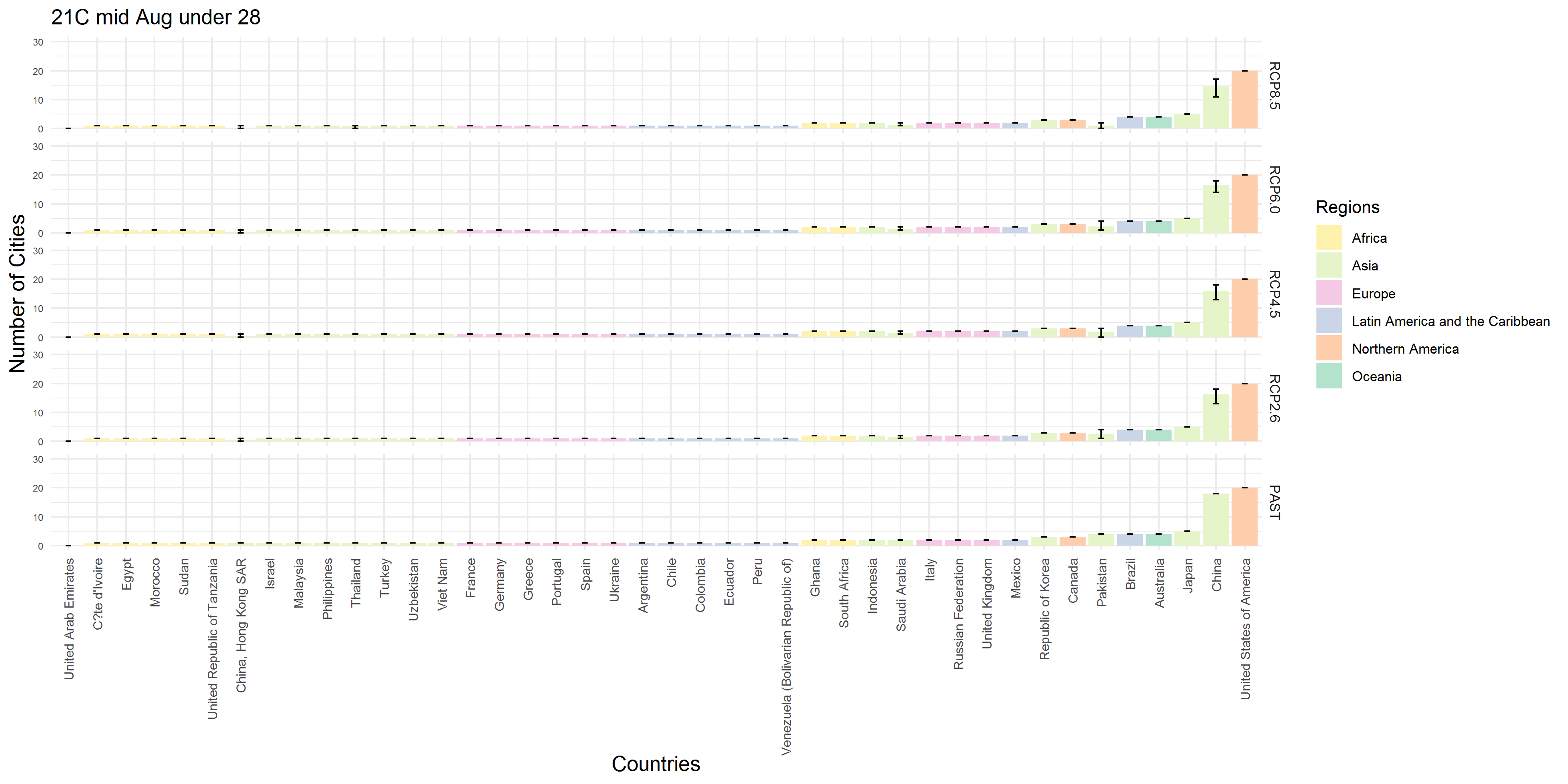  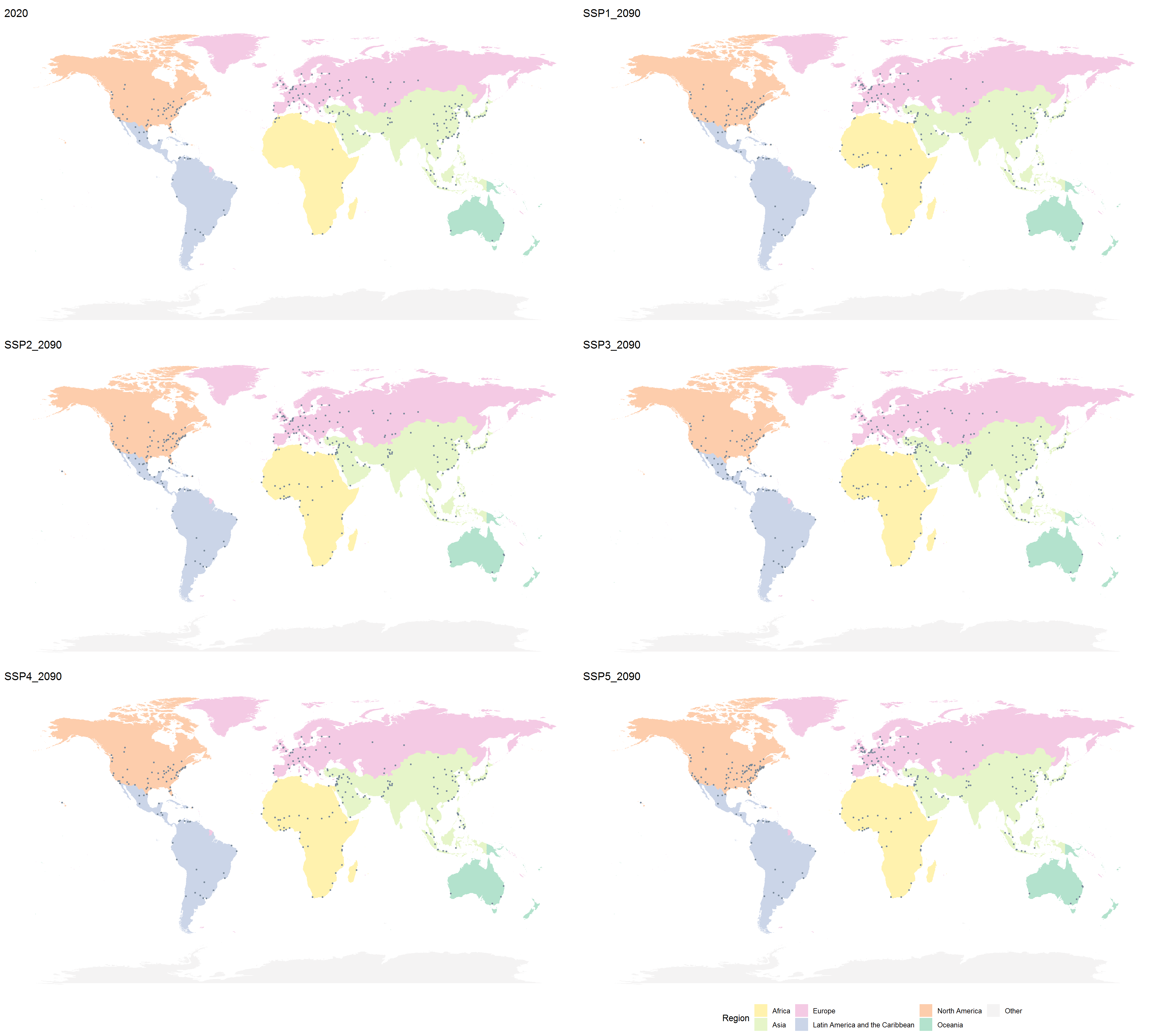  RCP: Representative Concentration Pathway |
| --- |

1. The number of cities that can host the Olympic marathon (WBGT levels 1 to 3) in the mid-21st century (2040–2059) under SSP1 by RCP/country. Note that the results are only for the cities covered in this study, and the distribution of the number of cities by country may be different if cities that do not publish meteorological data in the NOAA database are included. Error bars indicate the range between the maximum and minimum values of the seven GCMs.

| 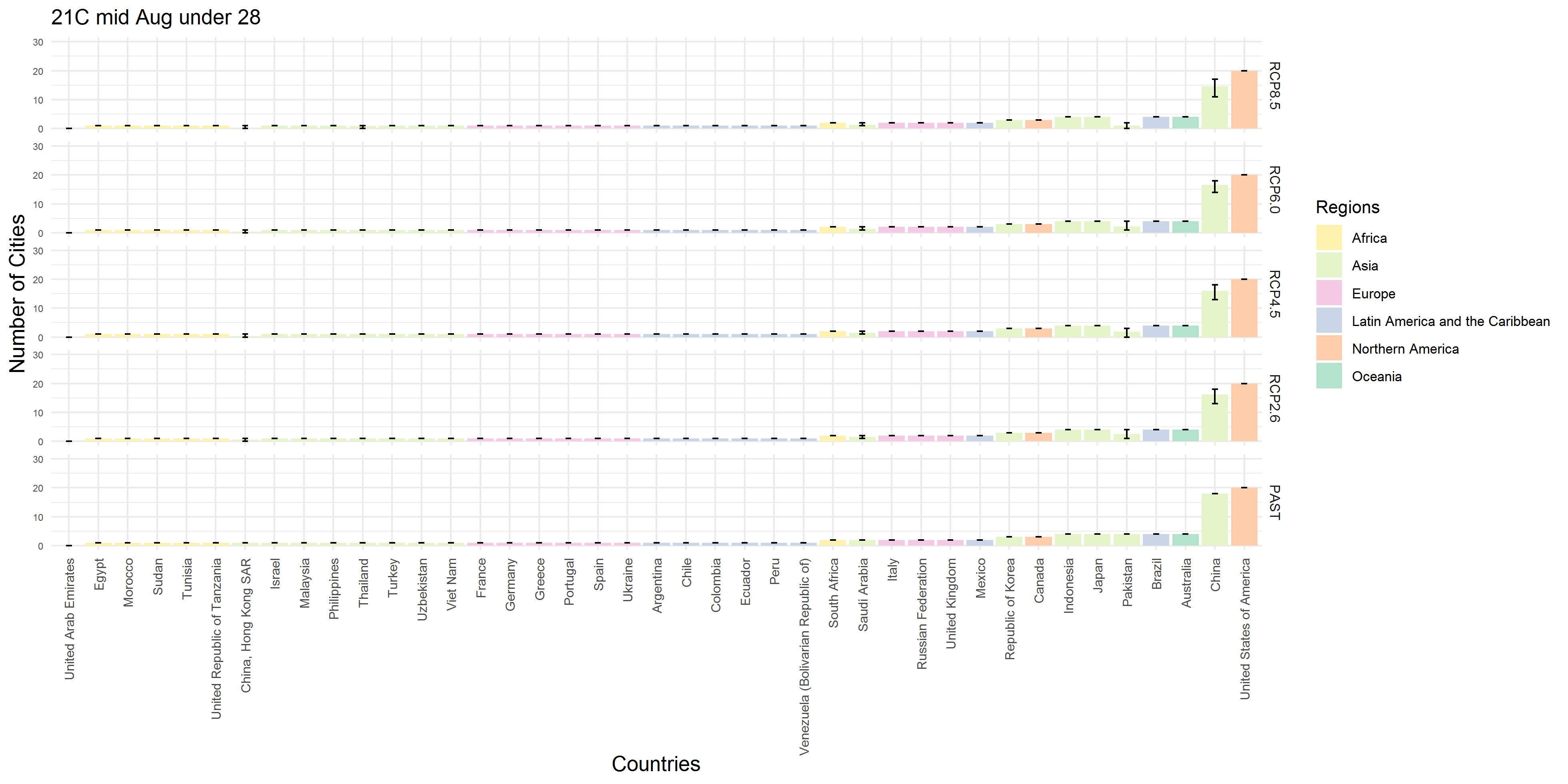  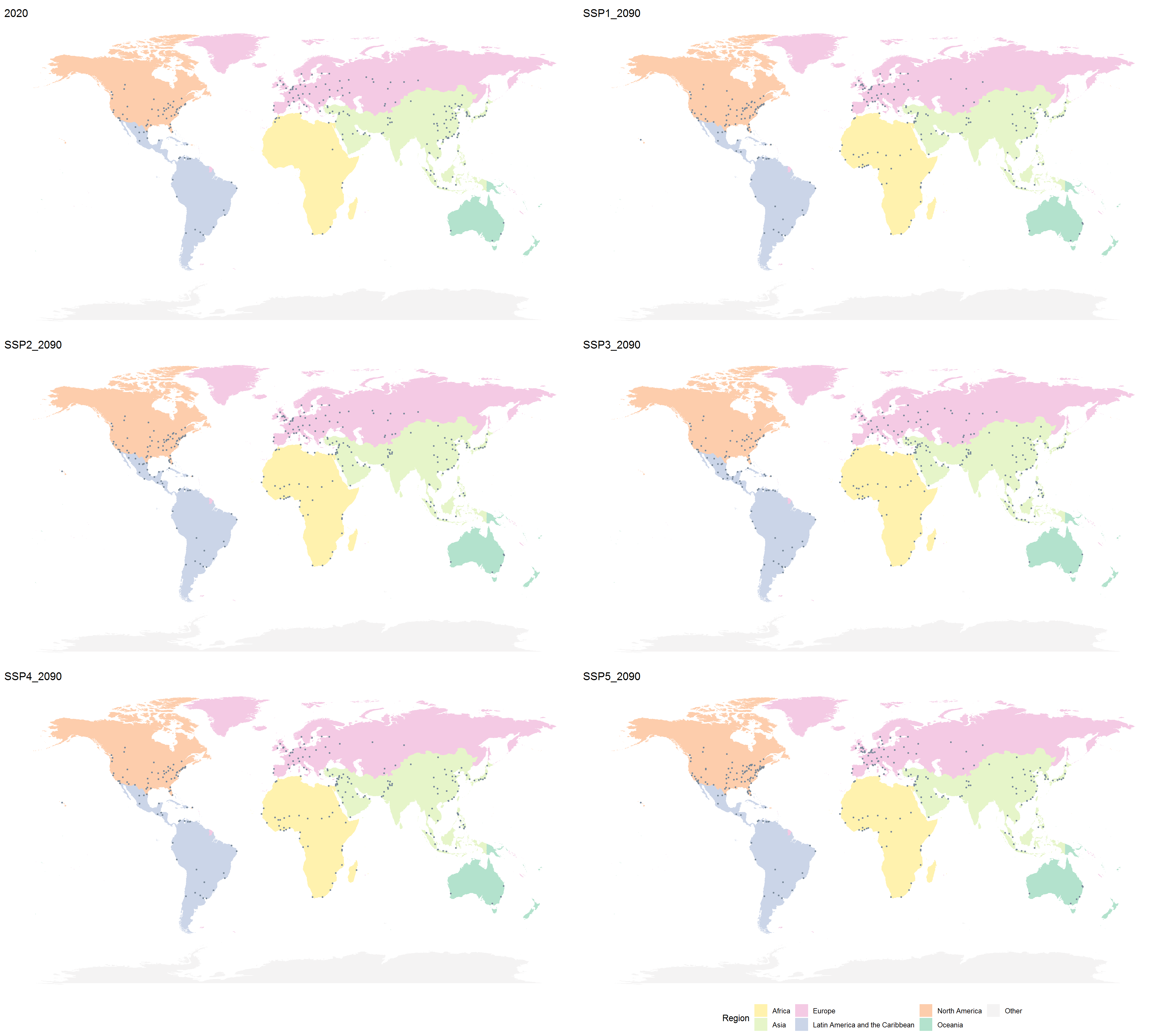  RCP: Representative Concentration Pathway |
| --- |

1. The number of cities that can host the Olympic marathon (WBGT levels 1 to 3) in the mid-21st century (2040–2059) under SSP2 by RCP/country. Note that the results are only for the cities covered in this study, and the distribution of the number of cities by country may be different if cities that do not publish meteorological data in the NOAA database are included. Error bars indicate the range between the maximum and minimum values of the seven GCMs.

| 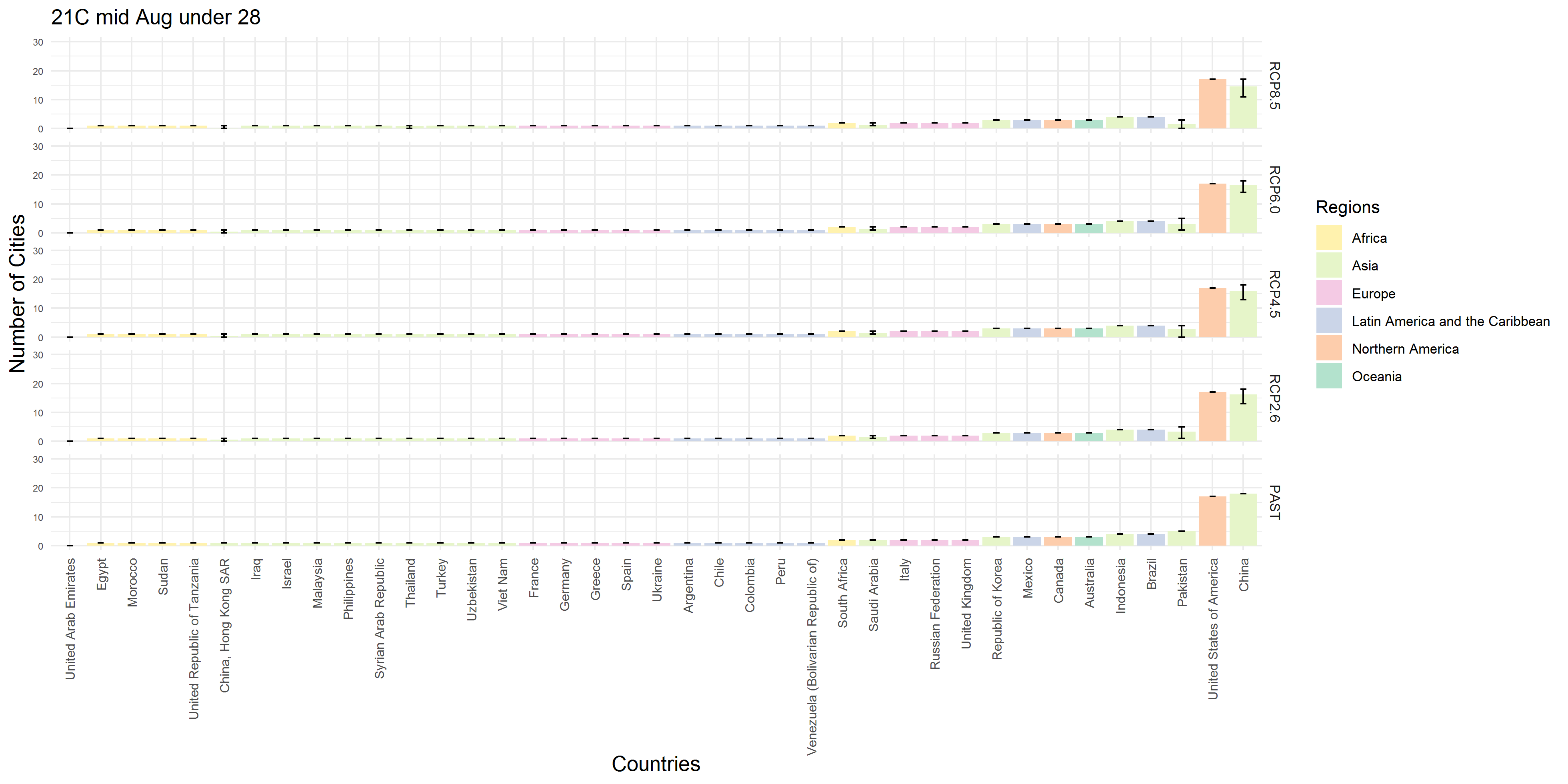  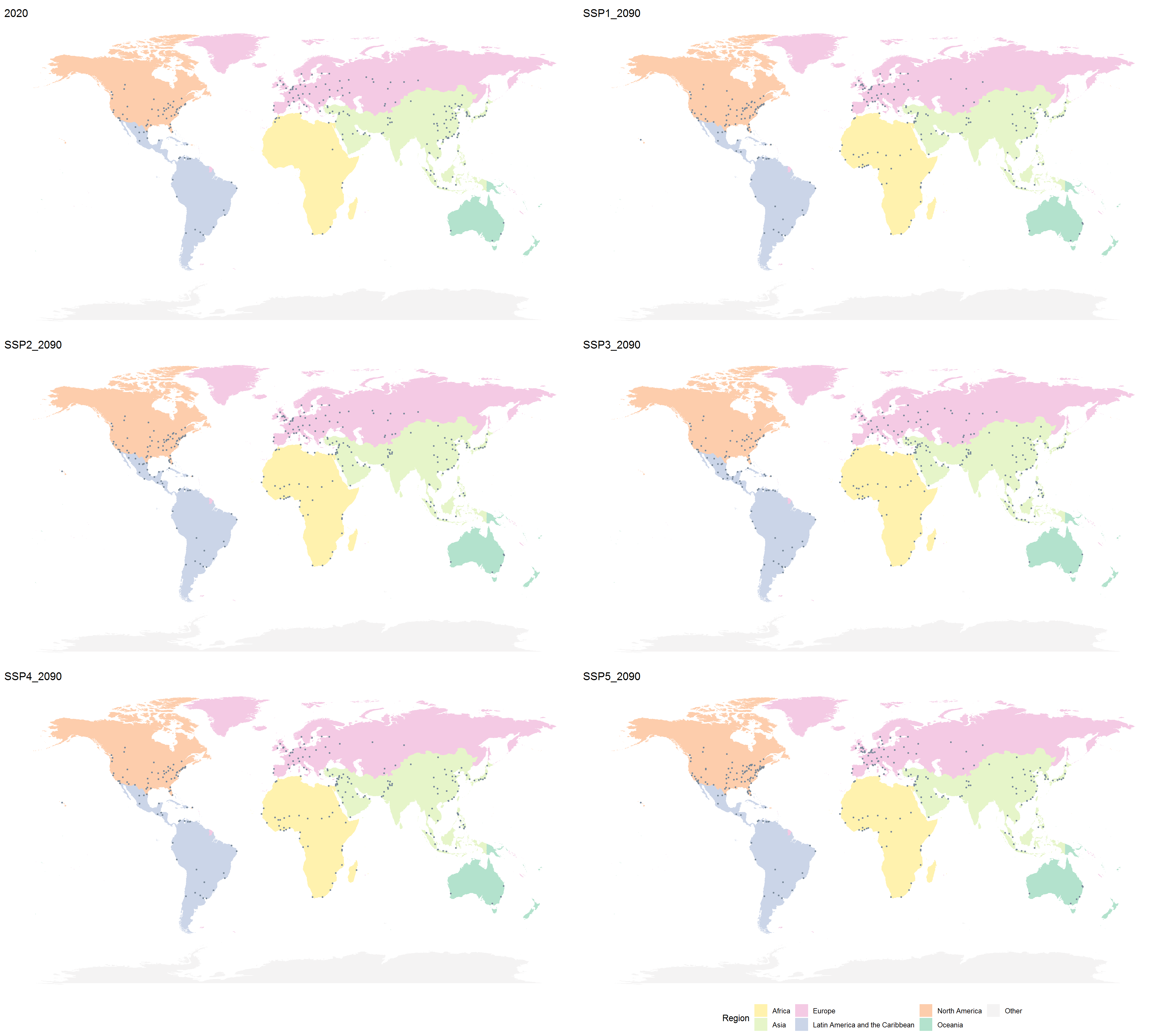  RCP: Representative Concentration Pathway |
| --- |

1. The number of cities that can host the Olympic marathon (WBGT levels 1 to 3) in the mid-21st century (2040–2059) under SSP3 by RCP/country. Note that the results are only for the cities covered in this study, and the distribution of the number of cities by country may be different if cities that do not publish meteorological data in the NOAA database are included. Error bars indicate the range between the maximum and minimum values of the seven GCMs.

| 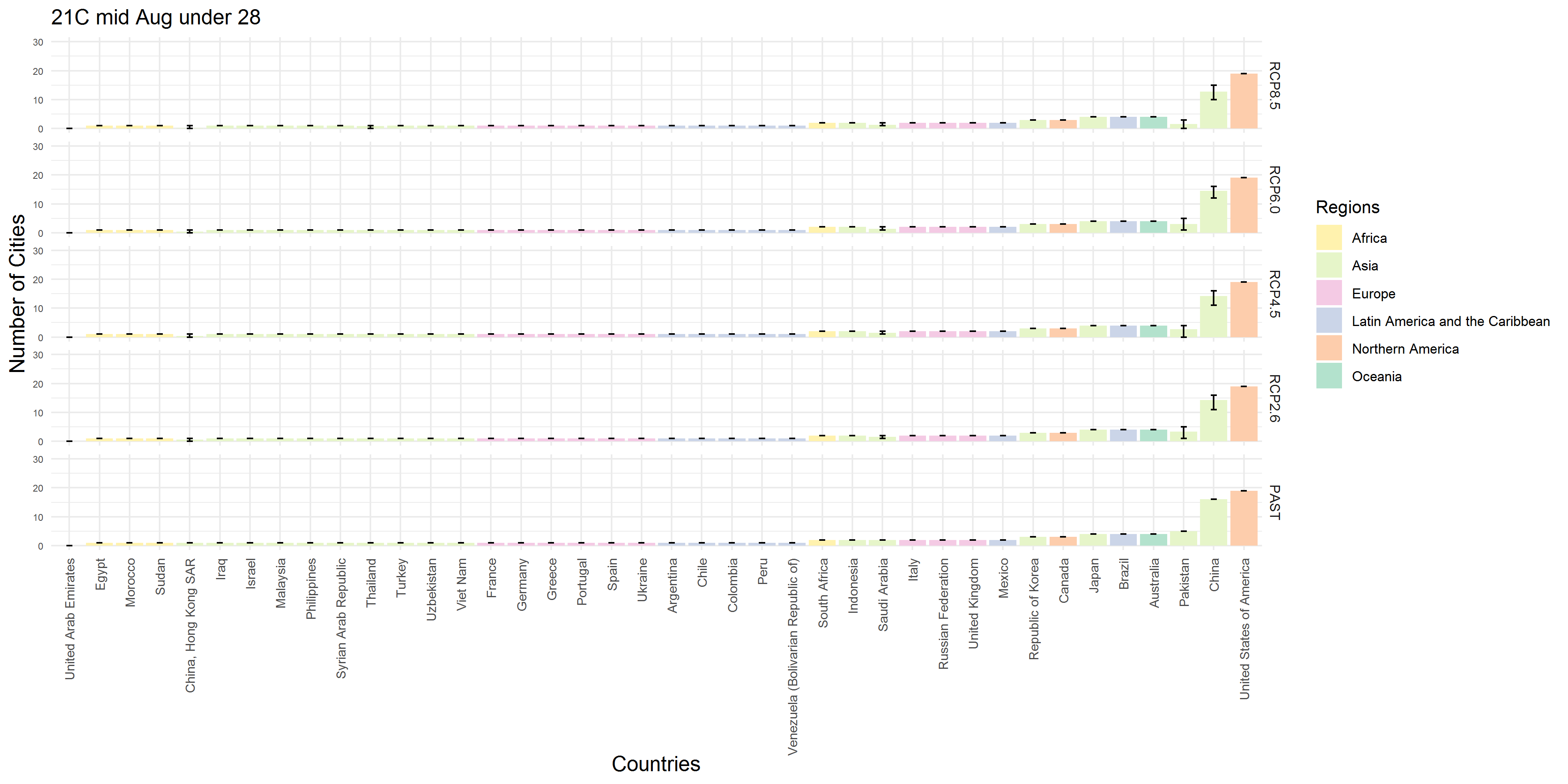  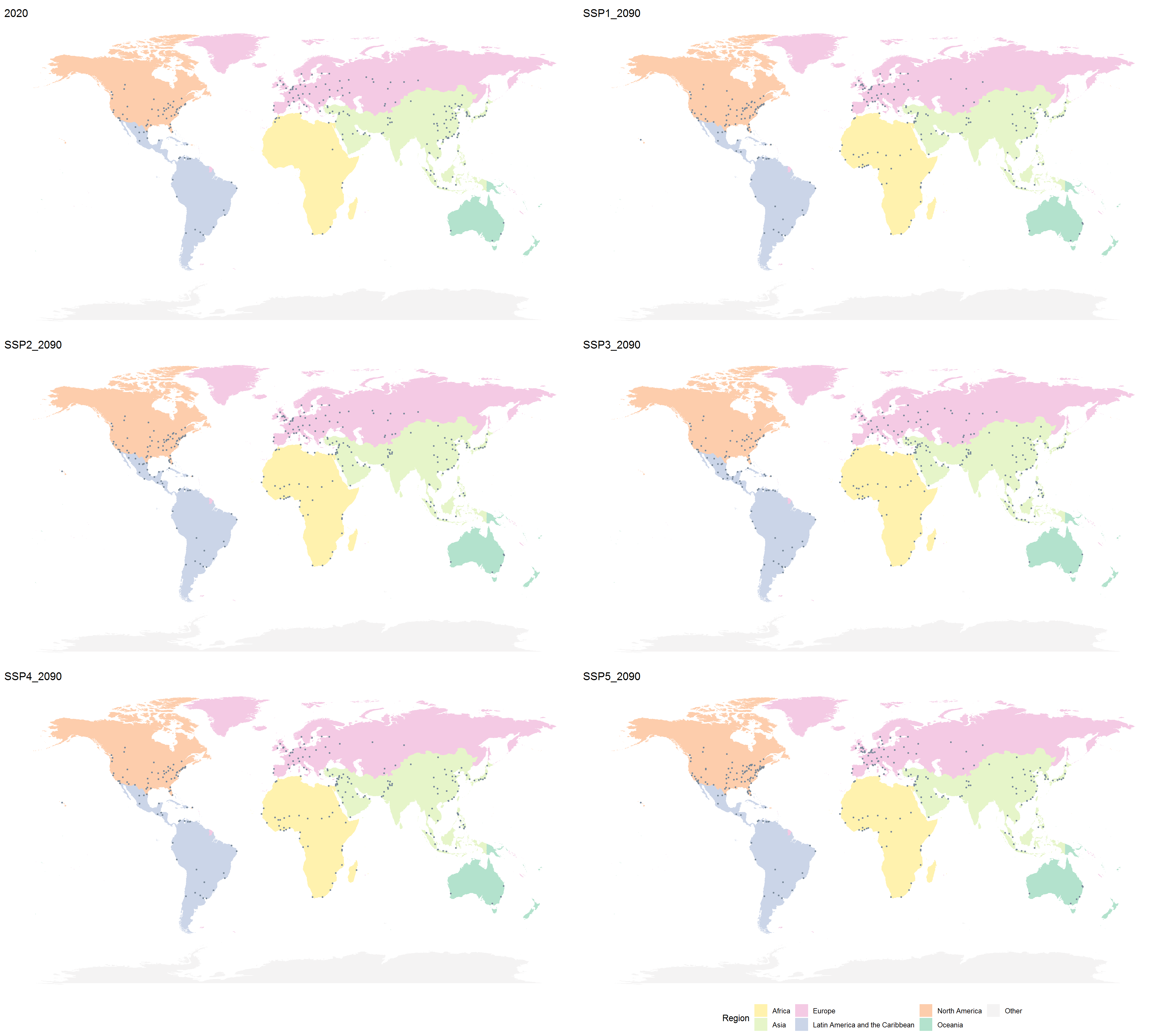  RCP: Representative Concentration Pathway |
| --- |

1. The number of cities that can host the Olympic marathon (WBGT levels 1 to 3) in the mid-21st century (2040–2059) under SSP4 by RCP/country. Note that the results are only for the cities covered in this study, and the distribution of the number of cities by country may be different if cities that do not publish meteorological data in the NOAA database are included. Error bars indicate the range between the maximum and minimum values of the seven GCMs.

| 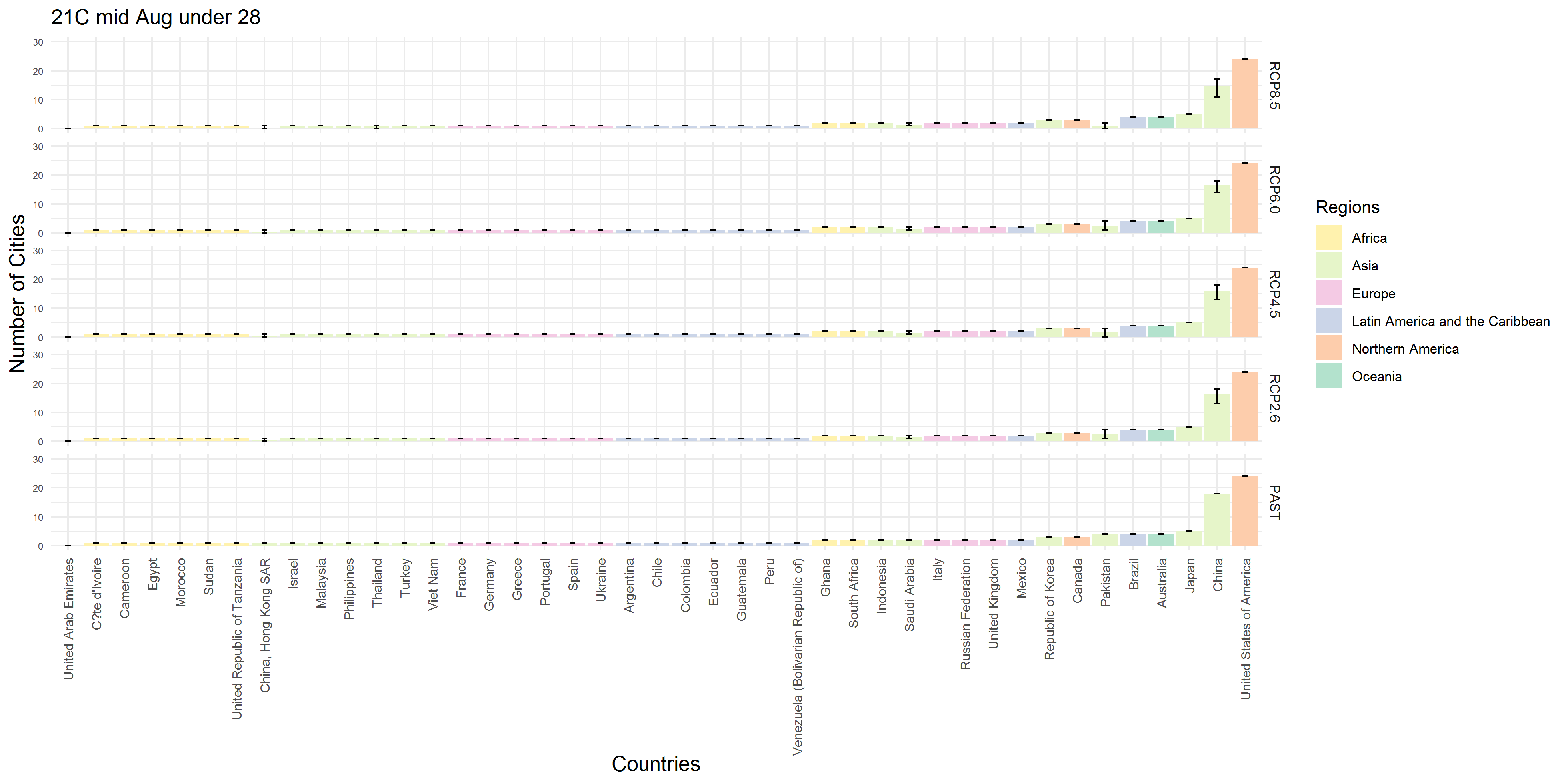  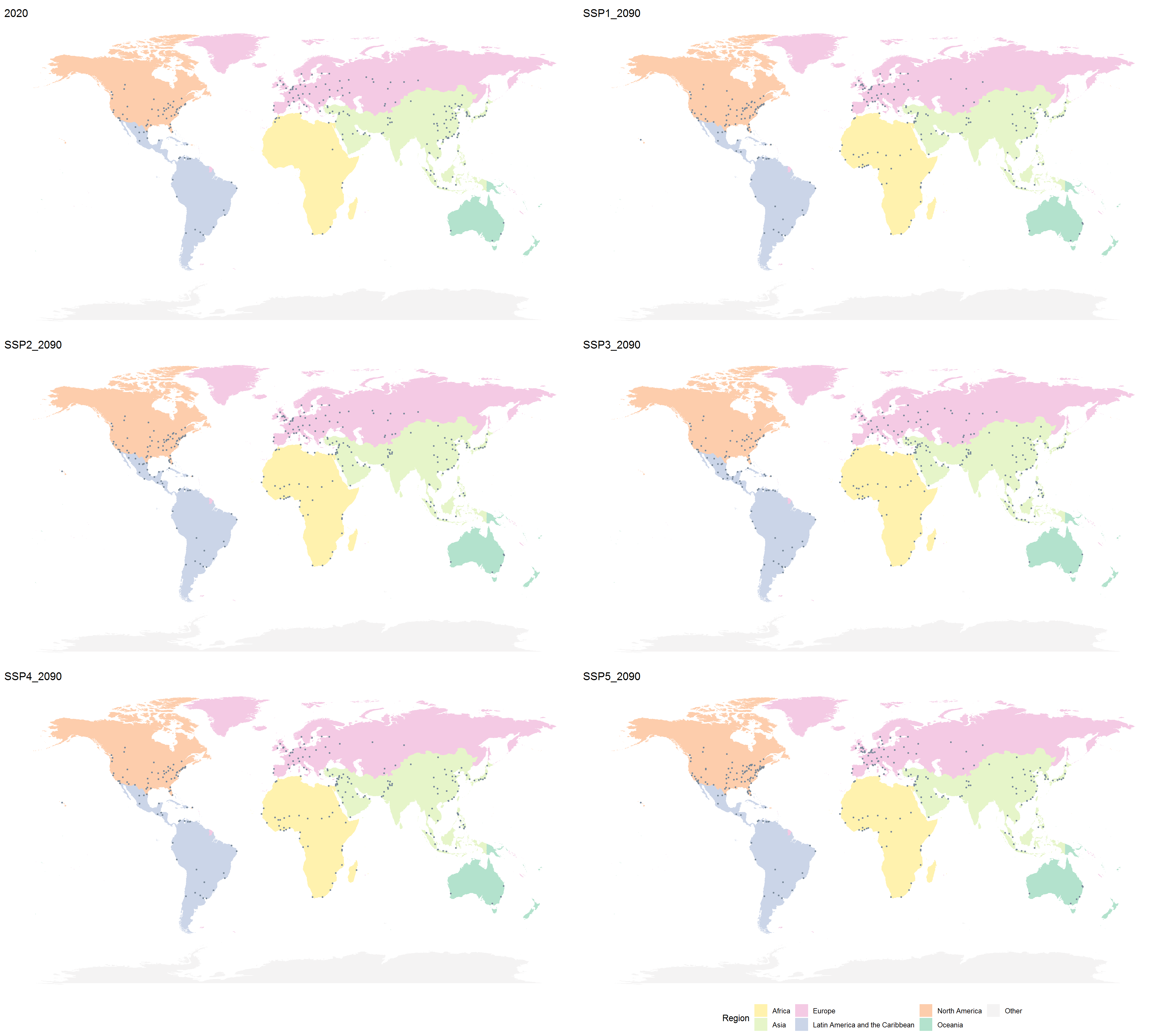  RCP: Representative Concentration Pathway |
| --- |

1. The number of cities that can host the Olympic marathon (WBGT levels 1 to 3) in the mid-21st century (2040–2059) under SSP5 by RCP/country. Note that the results are only for the cities covered in this study, and the distribution of the number of cities by country may be different if cities that do not publish meteorological data in the NOAA database are included. Error bars indicate the range between the maximum and minimum values of the seven GCMs.

| 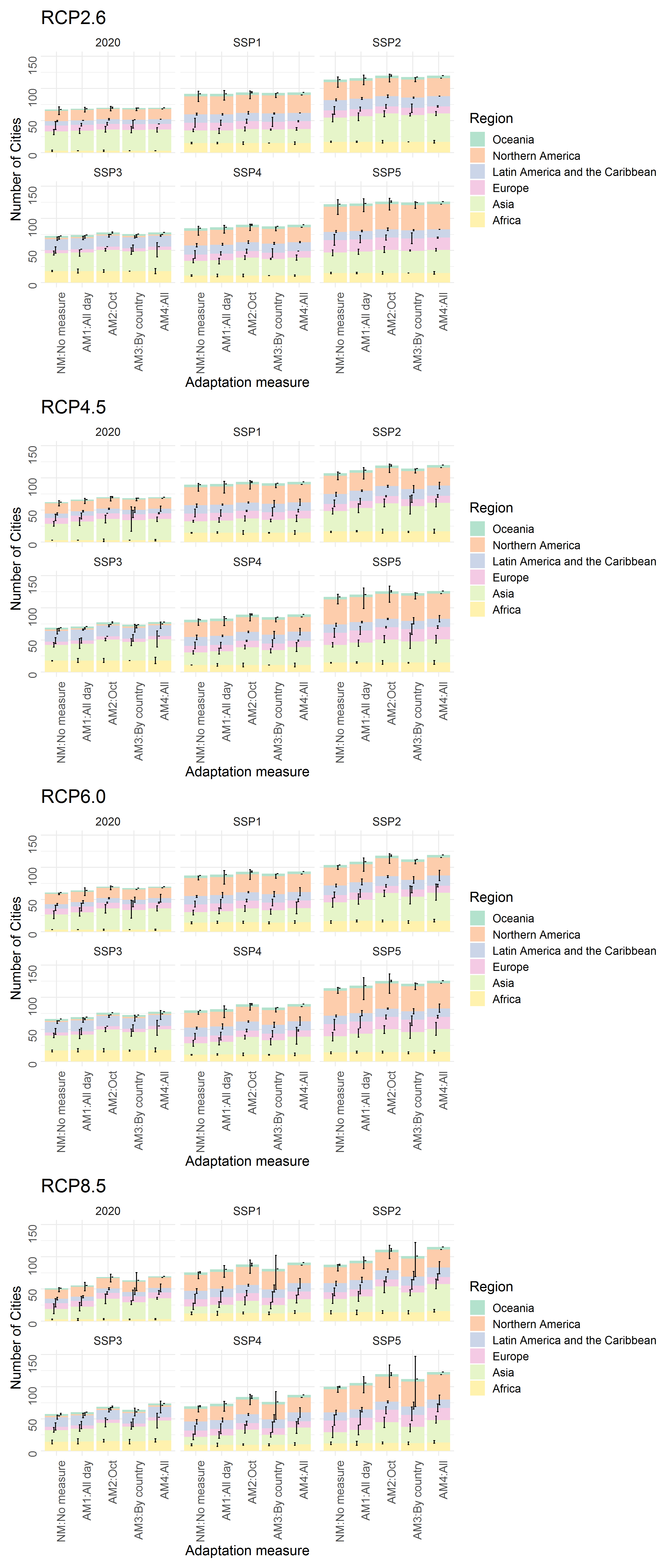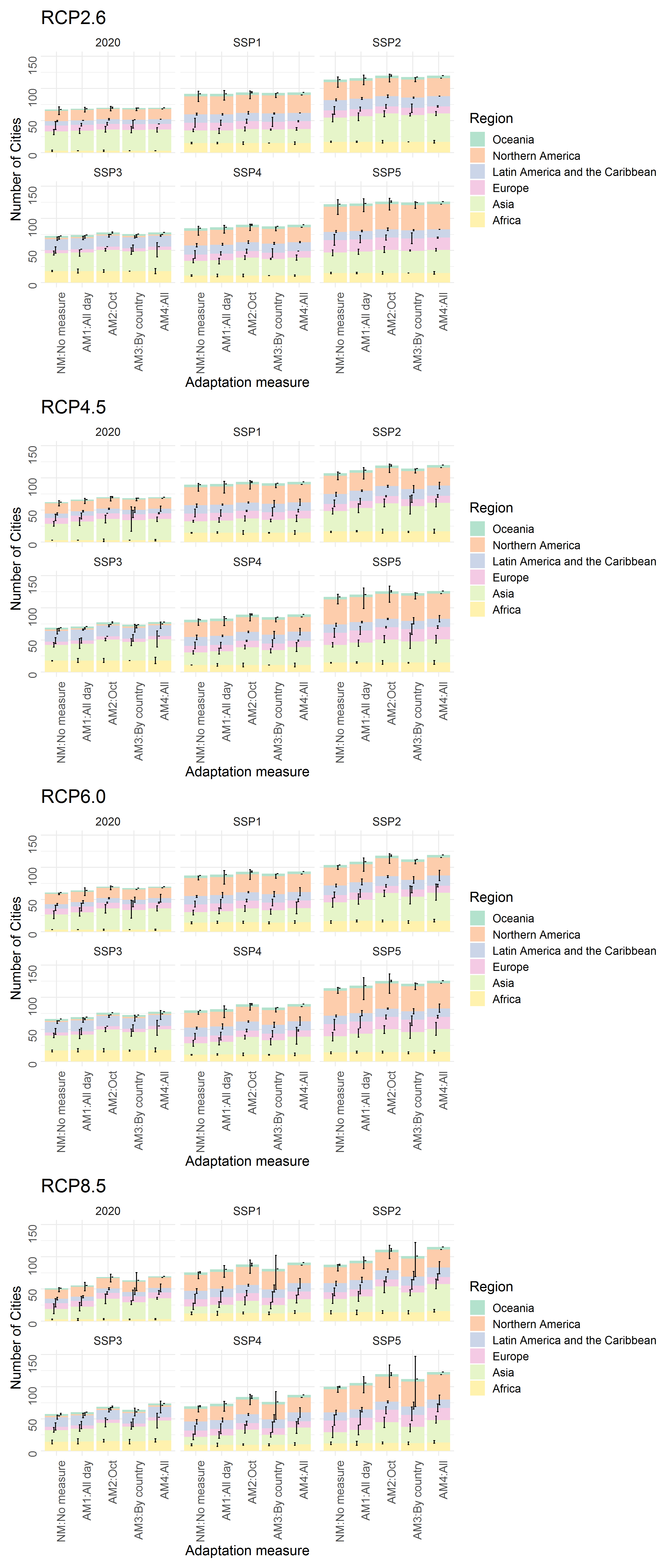 |
| --- |
| 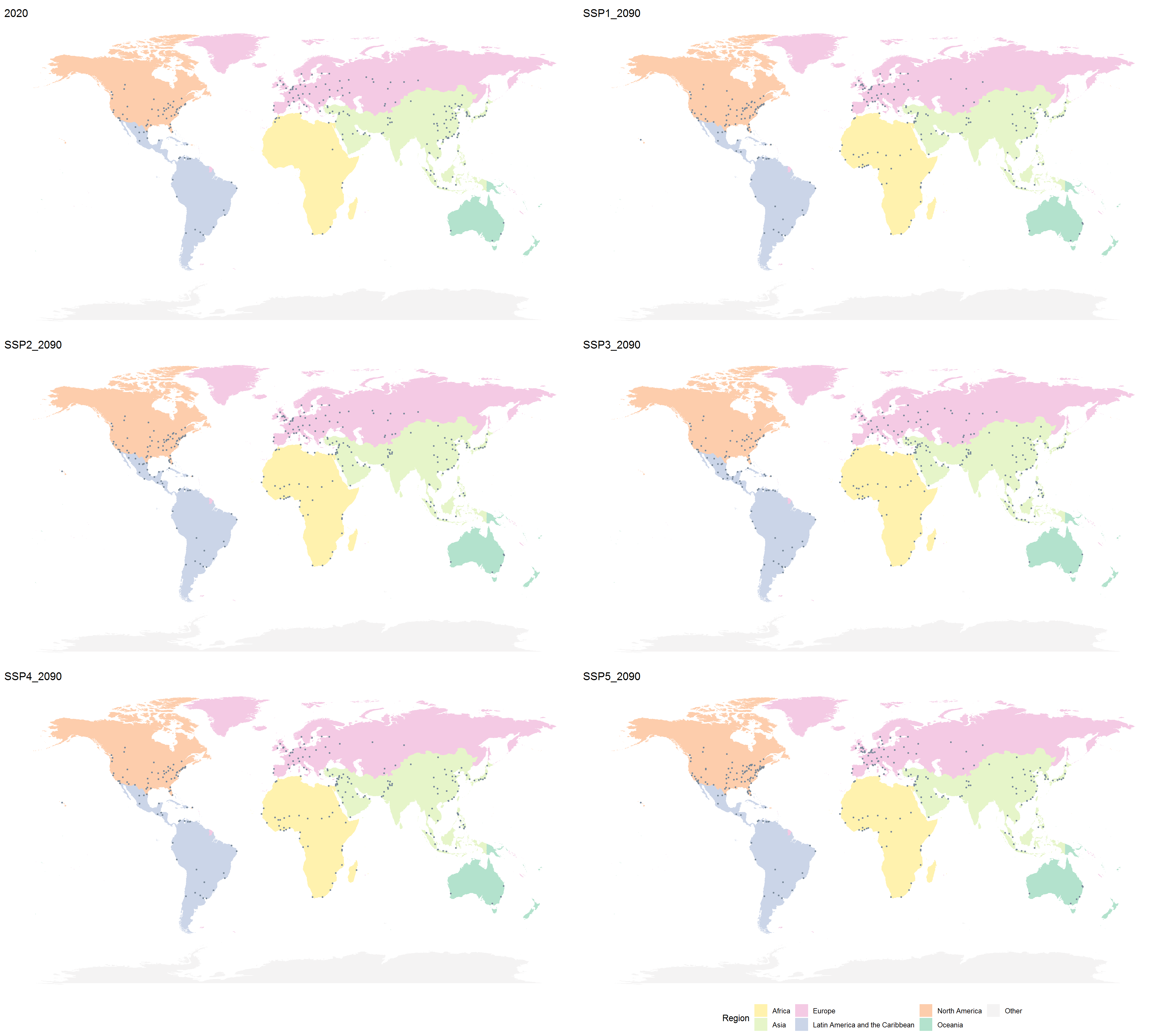  RCP: Representative Concentration Pathway / SSP: Shared Socioeconomic Pathway / AM: Adaptation Measure |

1. Change in the number of cities that can host the Olympic marathon (WBGT levels 1 to 3) due to adaptation measures in the late 21st century (2080–2099) by RCP/SSP/region. Error bars indicate the range between the maximum and minimum values of the seven GCMs.

| 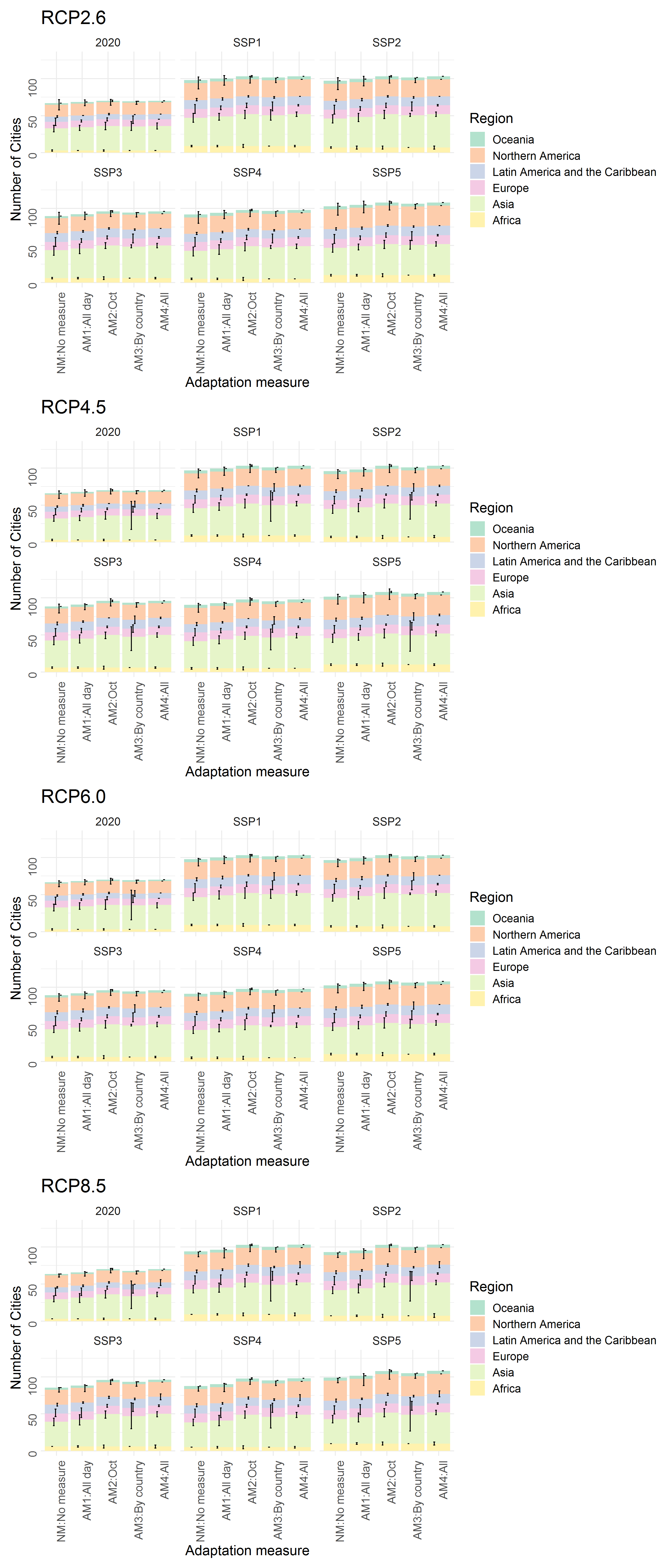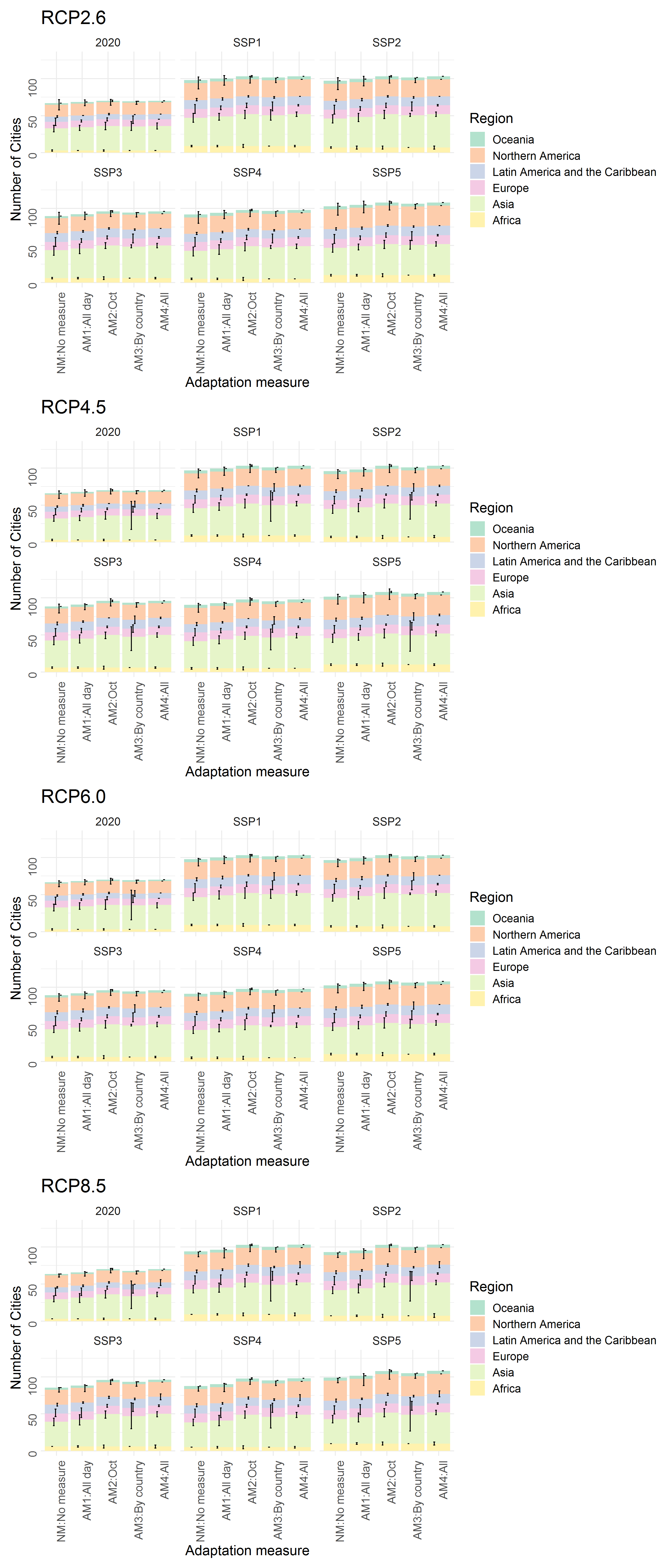  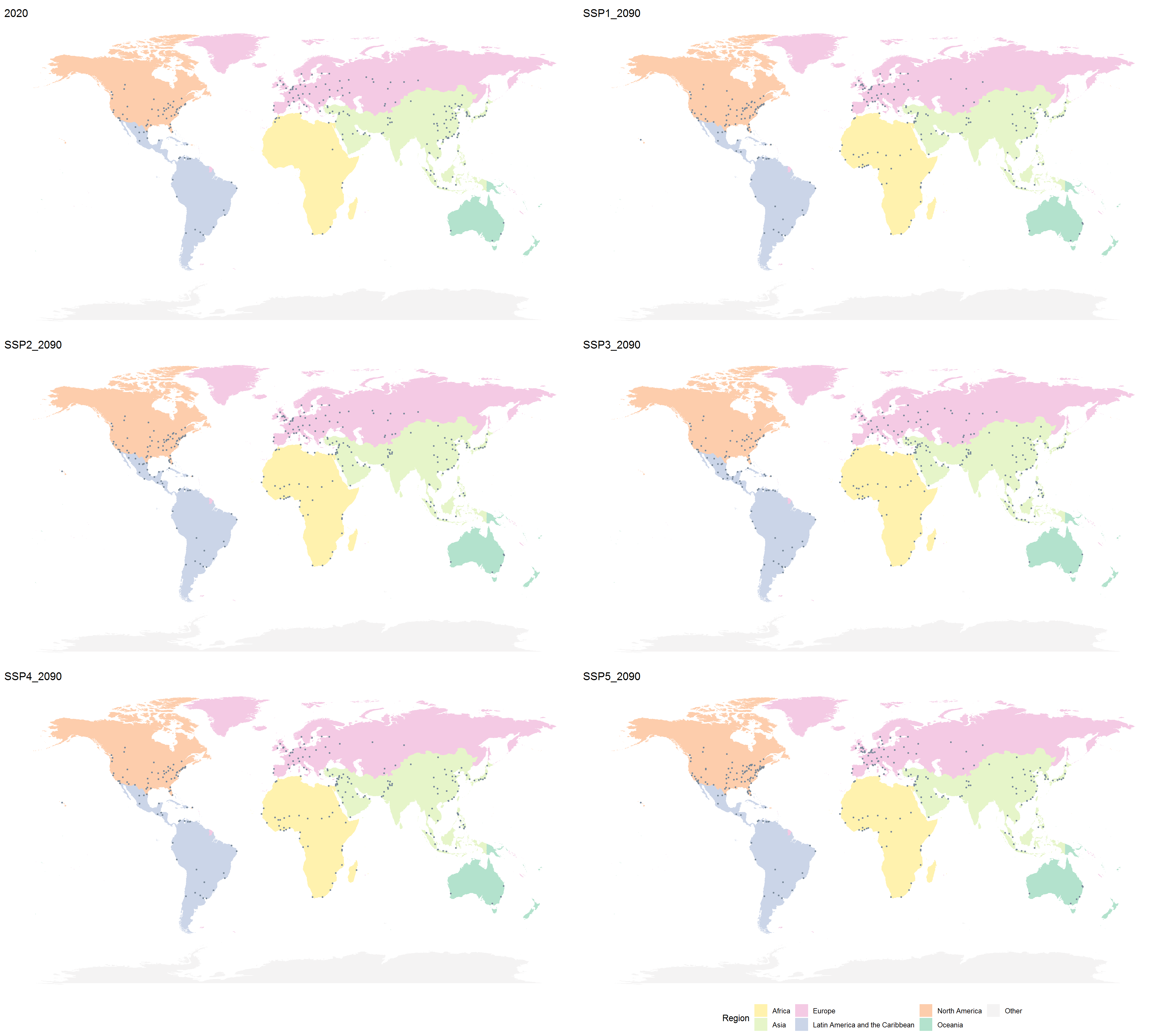  RCP: Representative Concentration Pathway / SSP: Shared Socioeconomic Pathway / AM: Adaptation Measure |
| --- |

1. Change in the number of cities that can host the Olympic marathon (WBGT levels 1 to 3) due to adaptation measures in the mid-21st century (2040–2059) by RCP/SSP/region. Error bars indicate the range between the maximum and minimum values of the seven GCMs.

| 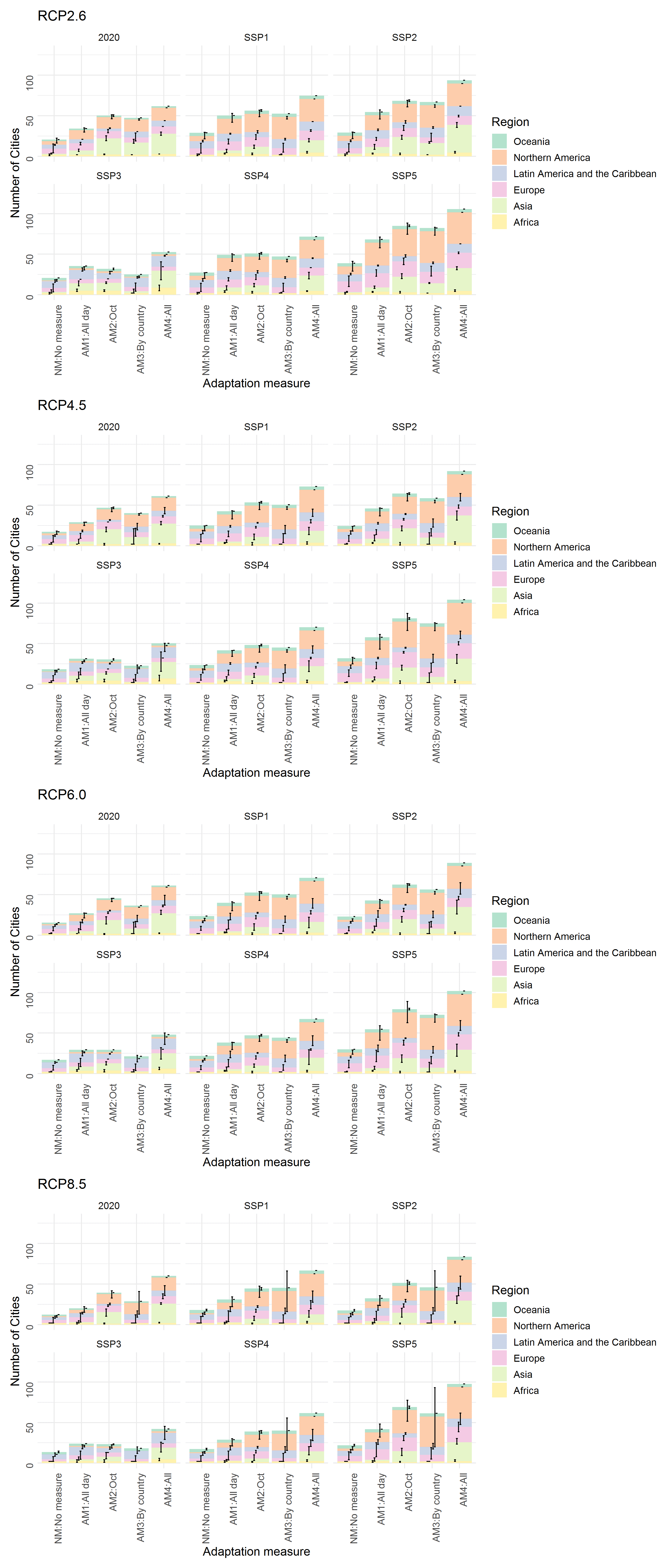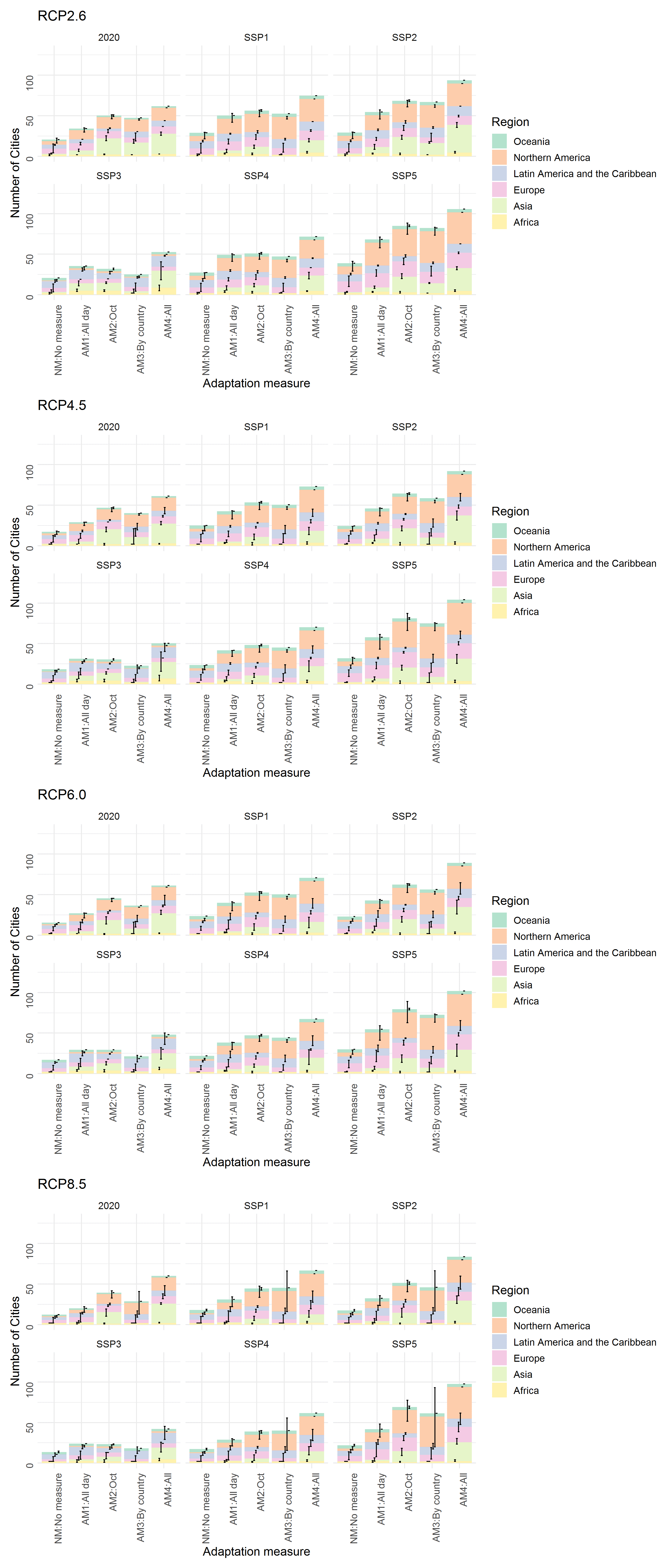  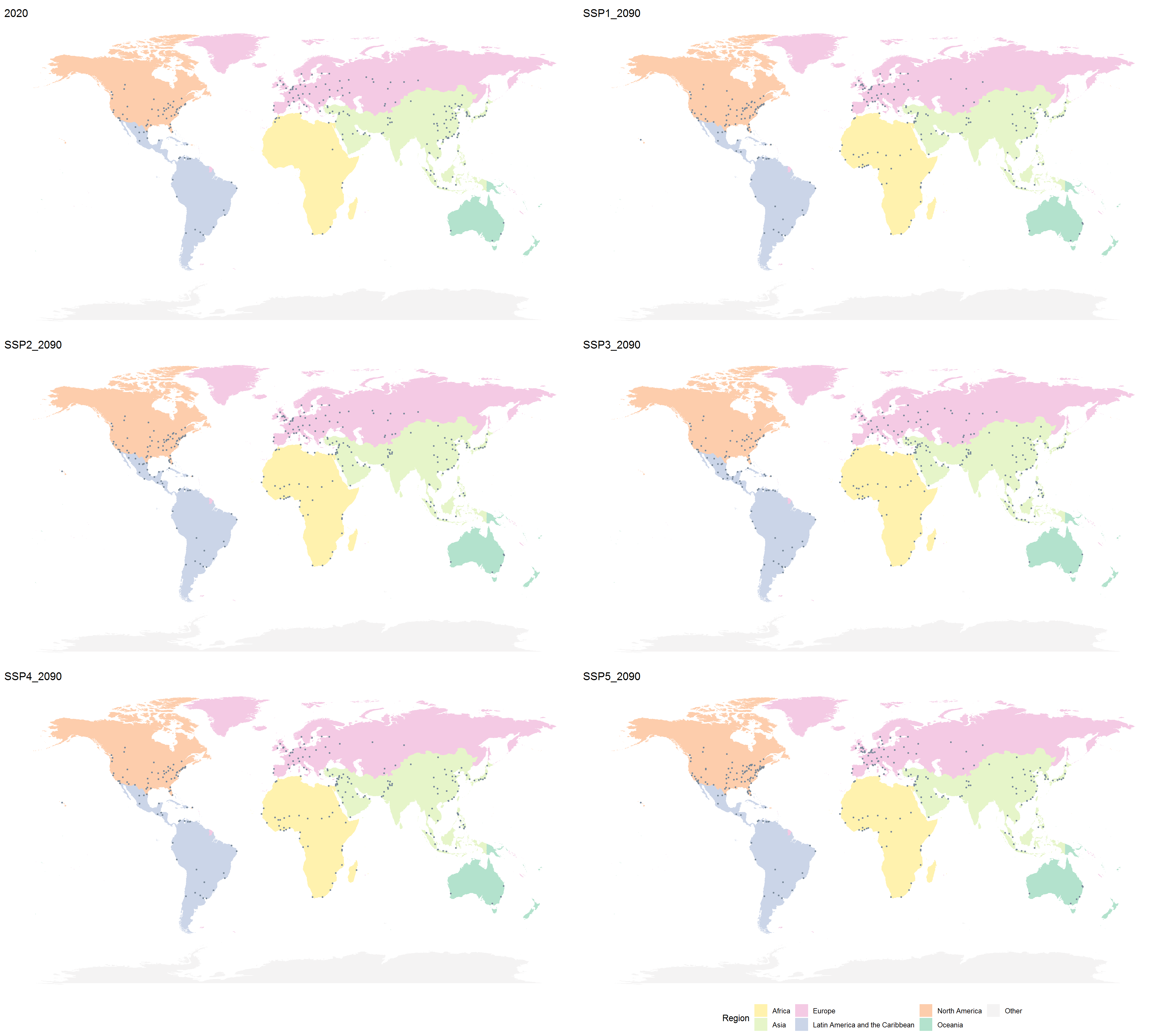  RCP: Representative Concentration Pathway / SSP: Shared Socioeconomic Pathway / AM: Adaptation Measure |
| --- |

1. Change in the number of cities that can host the Olympic marathon at low risk (WBGT levels 1 to 2) due to adaptation measures in the late 21st century (2080–2099) by RCP/SSP/region. Error bars indicate the range between the maximum and minimum values of the seven GCMs.

| 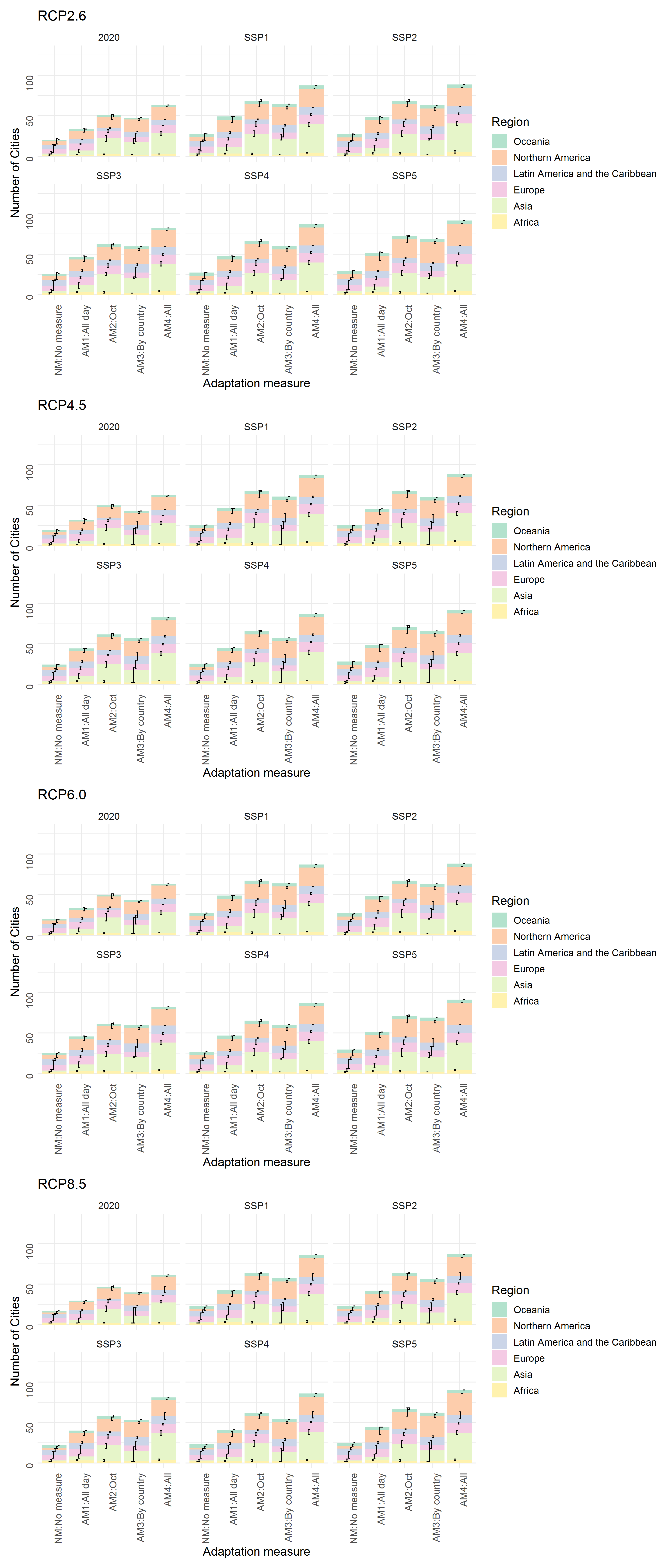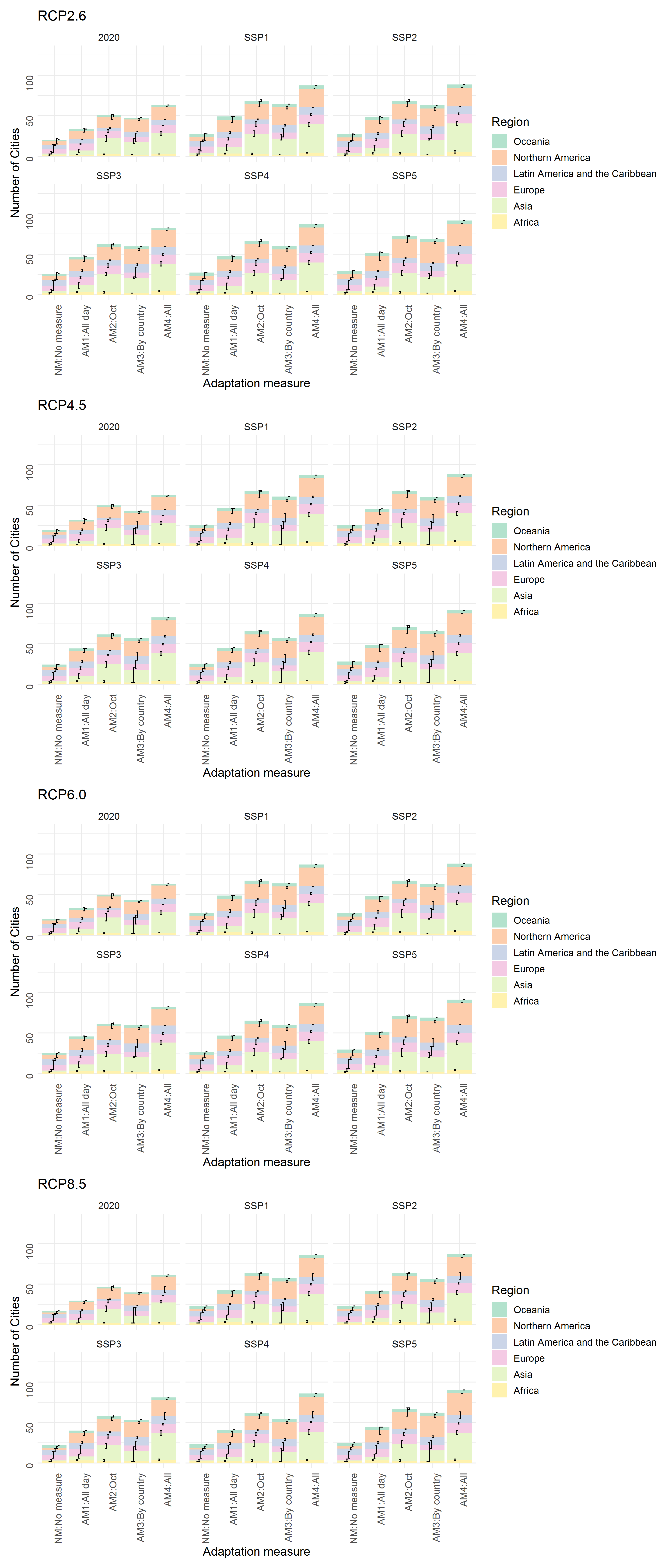  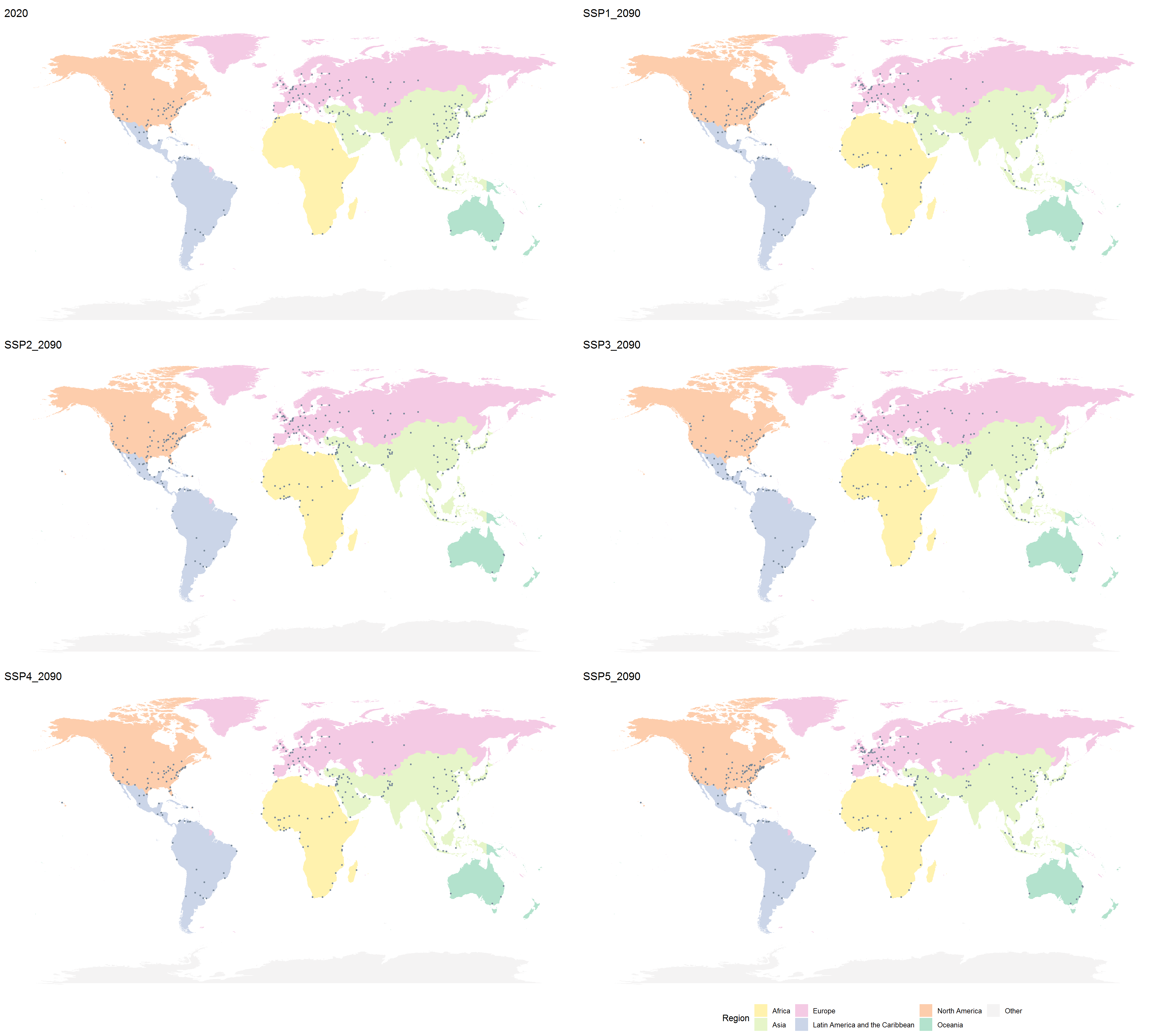  RCP: Representative Concentration Pathway / SSP: Shared Socioeconomic Pathway / AM: Adaptation Measure |
| --- |

1. Change in the number of cities that can host the Olympic marathon at low risk (WBGT levels 1 to 2) due to adaptation measures in the mid-21st century (2040–2059) by RCP/SSP/region. Error bars indicate the range between the maximum and minimum values of the seven GCMs.

|  |
| --- |

1. WBGT levels in 19 cities that have hosted and/or are scheduled to host the Games since 1948 (mid-21st century (2040–2059), RCP2.6–8.5, SSP1–5). The numbers in the columns under climate indicate the WBGT levels. In the columns under Society & Economy, “T” indicates that the city could be a candidate for hosting the Olympics under the socioeconomic conditions in 2010 or 2030–2050 (SSP1–5), while “F” indicates that the city is not a good candidate.

|  |
| --- |

1. WBGT levels in cities in Africa (late-21st century (2080–2099), RCP2.6–8.5, SSP1–5). The numbers in the columns under climate indicate the WBGT levels. In the columns under Society & Economy, “T” indicates that the city could be a candidate for hosting the Olympics under the socioeconomic conditions in 2010 or 2070–2090 (SSP1–5), while “F” indicates that the city is not a good candidate.

|    |
| --- |

1. WBGT levels in cities in Asia (late-21st century (2080–2099), RCP2.6–8.5, SSP1–5). The numbers in the columns under climate indicate the WBGT levels. In the columns under Society & Economy, “T” indicates that the city could be a candidate for hosting the Olympics under the socioeconomic conditions in 2010 or 2070–2090 (SSP1–5), while “F” indicates that the city is not a good candidate.

|    |
| --- |

1. WBGT levels in cities in Europe (late-21st century (2080–2099), RCP2.6–8.5, SSP1–5). The numbers in the columns under climate indicate the WBGT levels. In the columns under Society & Economy, “T” indicates that the city could be a candidate for hosting the Olympics under the socioeconomic conditions in 2010 or 2070–2090 (SSP1–5), while “F” indicates that the city is not a good candidate.

|    |
| --- |

1. WBGT levels in cities in Latin America and the Caribbean (late-21st century (2080–2099), RCP2.6–8.5, SSP1–5). The numbers in the columns under climate indicate the WBGT levels. In the columns under Society & Economy, “T” indicates that the city could be a candidate for hosting the Olympics under the socioeconomic conditions in 2010 or 2070–2090 (SSP1–5), while “F” indicates that the city is not a good candidate.

|    |
| --- |

1. WBGT levels in cities in Northern America (late-21st century (2080–2099), RCP2.6–8.5, SSP1–5). The numbers in the columns under climate indicate the WBGT levels. In the columns under Society & Economy, “T” indicates that the city could be a candidate for hosting the Olympics under the socioeconomic conditions in 2010 or 2070–2090 (SSP1–5), while “F” indicates that the city is not a good candidate.

|    |
| --- |

1. WBGT levels in cities in Oceania (late-21st century (2080–2099), RCP2.6–8.5, SSP1–5). The numbers in the columns under climate indicate the WBGT levels. In the columns under Society & Economy, “T” indicates that the city could be a candidate for hosting the Olympics under the socioeconomic conditions in 2010 or 2070–2090 (SSP1–5), while “F” indicates that the city is not a good candidate.

|  |
| --- |

1. WBGT levels in cities in Africa (mid-21st century (2040–2059), RCP2.6–8.5, SSP1–5). The numbers in the columns under climate indicate the WBGT levels. In the columns under Society & Economy, “T” indicates that the city could be a candidate for hosting the Olympics under the socioeconomic conditions in 2010 or 2030–2050 (SSP1–5), while “F” indicates that the city is not a good candidate.

|    |
| --- |

1. WBGT levels in cities in Asia (mid-21st century (2040–2059), RCP2.6–8.5, SSP1–5). The numbers in the columns under climate indicate the WBGT levels. In the columns under Society & Economy, “T” indicates that the city could be a candidate for hosting the Olympics under the socioeconomic conditions in 2010 or 2030–2050 (SSP1–5), while “F” indicates that the city is not a good candidate.

|    |
| --- |

1. WBGT levels in cities in Europe (mid-21st century (2040–2059), RCP2.6–8.5, SSP1–5). The numbers in the columns under climate indicate the WBGT levels. In the columns under Society & Economy, “T” indicates that the city could be a candidate for hosting the Olympics under the socioeconomic conditions in 2010 or 2030–2050 (SSP1–5), while “F” indicates that the city is not a good candidate.

|    |
| --- |

1. WBGT levels in cities in Latin America and the Caribbean (mid-21st century (2040–2059), RCP2.6–8.5, SSP1–5). The numbers in the columns under climate indicate the WBGT levels. In the columns under Society & Economy, “T” indicates that the city could be a candidate for hosting the Olympics under the socioeconomic conditions in 2010 or 2030–2050 (SSP1–5), while “F” indicates that the city is not a good candidate.

|    |
| --- |

1. WBGT levels in cities in Northern America (mid-21st century (2040–2059), RCP2.6–8.5, SSP1–5). The numbers in the columns under climate indicate the WBGT levels. In the columns under Society & Economy, “T” indicates that the city could be a candidate for hosting the Olympics under the socioeconomic conditions in 2010 or 2030–2050 (SSP1–5), while “F” indicates that the city is not a good candidate.

|    |
| --- |

1. WBGT levels in cities in Oceania (mid-21st century (2040–2059), RCP2.6–8.5, SSP1–5). The numbers in the columns under climate indicate the WBGT levels. In the columns under Society & Economy, “T” indicates that the city could be a candidate for hosting the Olympics under the socioeconomic conditions in 2010 or 2030–2050 (SSP1–5), while “F” indicates that the city is not a good candidate.

|    |
| --- |

1. WBGT time trends in 19 cities that have hosted and/or are scheduled to host the Games since 1948 (August, the late-21st century, RCP2.6–8.5). Mexico City is excluded because it is located at an altitude of more than 1600 m, which is the target of this study's evaluation.

|  |
| --- |

1. Framework to estimate the WBGT. Rectangles represent the data, and rectangles with rounded corners represent processes. This framework was built based on Takakura et al. 2019, and the elements with red borders were added in this study. The following explanation of the framework is quoted from Takakura et al. 2019 and partially modified by the authors.

A statistical downscaling method for reconstructing hourly resolution WBGT from daily data was developed in previous studies (Takakura et al. 2017, 2018). Based on the statistical relationship between the daily climate variables and hourly WBGT, this method can produce hourly resolution WBGT taking daily representative climate variables as input using a statistical machine learning technique (Fan et al. 2008) as follows.


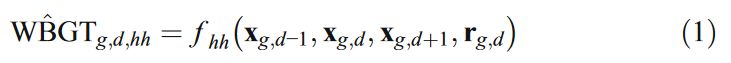


Here, x_g,d_=(x_g,d,1_, x_g,d,2_, ...,x_g,d,7_), where x_g,d,1_...,x_g,d,7_ are the daily mean temperature, daily maximum temperature, daily minimum temperature, daily mean relative humidity, daily mean wind speed, daily mean air pressure, and daily mean solar radiation in grid cell g on day d, respectively, and d−1 and d+1 represent the day before and after d, respectively. r_g,d_=(r_g,d,00_, r_g,d,01_,...,r_g,d,23_), and r_g,d,hh_=x_g,d,7_cosθ_g,d,hh_,whereθ_g,d,hh_ is the solar zenith angle in grid cell g at time hh on day d (if cosθ_g,d,hh_<0, r_g,d,hh_=0).These input variables were selected with consideration of their availability in the bias-corrected output of the GCMs (Hempel et al.2013; Iizumi et al.2017) and possible relationships to the hourly WBGT. For more details on this method, please see the supporting information of Takakura et al. 2019 and Takakura et al. 2018.

In the following, we denote the measured values as WBGT_s,d, hh_. The subscript s represents the site, and the corresponding grid cell that contains site s is denoted as g(s). At the same time, we obtain the reconstructed W^BGT_(g(s), d, hh)_ values based on the corresponding grid-based data (in this study, we use S14FD) using Eq. (1). Then, based on these two values, we calculate the errors(biases) in the reconstructed WBGT as follows.


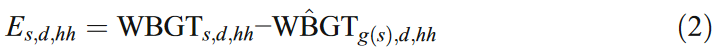


Then, we derive a formula to estimate E_s,d, hh_. This can be achieved by a simple linear regression model with two parametersα_s, hh_ andβ_s, hh_. The coefficientsα_s, hh_ andβ_s, hh_ can be calculated to minimize the value of Eq. (3), and this minimization problem can be solved analytically.


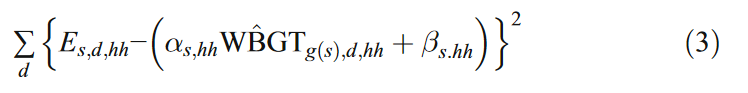


Finally, we obtain estimates for the site-specific, hourly resolution WBGT with the following formula.


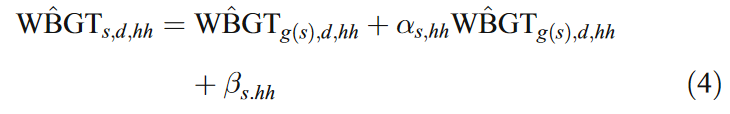

Supplement: Supplementary file 1 — Supplementary Information. [file 41598_2022_7934_MOESM1_ESM.docx]
